# Sample Size Requirements for Applying Mixed Polytomous Item Response Models: Results of a Monte Carlo Simulation Study

**DOI:** 10.3389/fpsyg.2019.02494

**Published:** 2019-11-13

**Authors:** Tanja Kutscher, Michael Eid, Claudia Crayen

**Affiliations:** ^1^Department of Education and Psychology, Freie Universitaet Berlin, Berlin, Germany; ^2^Department of Data Center and Method Development, Leibniz Institute for Educational Trajectories, Bamberg, Germany

**Keywords:** mixture IRT models, rating scale, sample size, model selection, Monte Carlo simulation

## Abstract

Mixture models of item response theory (IRT) can be used to detect inappropriate category use. Data collected by panel surveys where attitudes and traits are typically assessed by short scales with many response categories are prone to response styles indicating inappropriate category use. However, the application of mixed IRT models to this data type can be challenging because of many threshold parameters within items. Up to now, there is very limited knowledge about the sample size required for an appropriate performance of estimation methods as well as goodness-of-fit criteria of mixed IRT models in this case. The present Monte Carlo simulation study examined these issues for two mixed IRT models [the restricted mixed generalized partial credit model (rmGPCM) and the mixed partial credit model (mPCM)]. The population parameters of the simulation study were taken from a real application to survey data which is challenging (a 5-item scale with an 11-point rating scale, and three latent classes). Additional data conditions (e.g., long tests, a reduced number of response categories, and a simple latent mixture) were included in this simulation study to improve the generalizability of the results. Under this challenging data condition, for each model, data were generated based on varying sample sizes (from 500 to 5,000 observations with a 500-step). For the additional conditions, only three sample sizes (consisting of 1,000, 2,500, and 4,500 observations) were examined. The effect of sample size on estimation problems and accuracy of parameter and standard error estimates were evaluated. Results show that the two mixed IRT models require at least 2,500 observations to provide accurate parameter and standard error estimates under the challenging data condition. The rmGPCM produces more estimation problems than the more parsimonious mPCM, mostly because of the sparse tables arising due to many response categories. These models exhibit similar trends of estimation accuracy across sample sizes. Under the additional conditions, no estimation problems are observed. Both models perform well with a smaller sample size when long tests were used or a true latent mixture includes two classes. For model selection, the AIC3 and the SABIC are the most reliable information criteria.

## Introduction

Mixture models of item response theory (IRT) are a combination of IRT models and latent class analysis (see for an overview von Davier and Carstensen, 2007). They allow classifying individuals into homogeneous subpopulations that are a priori unknown (latent classes) and differ in the category characteristic curves linking the response probabilities with the latent trait variable (Rost, 1997). The mixture IRT approach for polytomous items is widely applied in empirical social research, mainly with the purpose of exploring population heterogeneity and its causes. For example, mixture polytomous IRT models are useful for detecting latent classes that qualitatively differ in a measured personality trait or attitude (e.g., Egberink et al., 2010; Finch and Pierson, 2011; Baghaei and Carstensen, 2013; Gnaldi et al., 2016; Jensuttiwetchakul et al., 2016) or those that are characterized by response styles (e.g., Eid and Rauber, 2000; Austin et al., 2006; Wagner-Menghin, 2006; Eid and Zickar, 2007; Maij-de Meij et al., 2008; Meiser and Machunsky, 2008; Wu and Huang, 2010; Wetzel et al., 2013). Moreover, they can be applied to examine construct validity (e.g., von Davier and Yamamoto, 2007; Tietjens et al., 2012), to detect differential item functioning (e.g., Frick et al., 2015; Cho et al., 2016), and to check the quality of a rating scale in general (e.g., Smith et al., 2011; Kutscher et al., 2017).

Compared to other statistical techniques that have been used to assess and control inappropriate category use (see for overview Van Vaerenbergh and Thomas, 2013), a distinguished advantage of the mixture IRT approach is that it can successfully represent different types of category use patterns (response styles) in one model. Mixed IRT models have been applied to detect response styles such as the preferences for extreme categories (ERS) or for middle categories (MRS), faking or socially desirable responding (e.g., Ziegler and Kemper, 2013; Mneimneh et al., 2014), and skipping superfluous response categories (e.g., Smith et al., 2011; Kutscher et al., 2017). Moreover, the application of a mixed IRT model does not require an a priori idea about the types of category use that might exist in the data, a single response style definition or an additional set of (heterogeneous) items in the questionnaire in order to measure response styles. Category use patterns are interpreted a posteriori based on the estimated class-specific item parameters. Due to their parsimony, the mixed partial credit model (mPCM; Rost, 1997) has been most often applied to explore category use in diverse research contexts (e.g., see Meiser and Machunsky, 2008; Wu and Huang, 2010; Jasper et al., 2013). The assumption of equally discriminating items can be considered a disadvantage of the mPCM, because such data can hardly be observed in empirical reality, and if not met, such a restriction increases the probability of identifying a wrong number of latent classes (Alexeev et al., 2011). Alternatively, the mixture extensions of multi-parameter IRT models [e.g., the generalized partial credit model (GPCM; Muraki, 1997) or the normal response model (NRM; Bock, 1972)] are more flexible und show a better fit to real-world data by including freely estimated discrimination parameters of items or categories (van der Linden and Hambleton, 1997). Only few studies applied any of the latter group of models for exploring category use (see Maij-de Meij et al., 2008; Egberink et al., 2010; Kutscher et al., 2017). The hesistance to apply these models might partly be due to the lack of systematic research on the performance of complex mixture IRT models under various data situations (Embretson and Reise, 2013). For example, it is unclear whether an application of a complex mixed IRT model would require a larger sample size or cause more estimation problems than a more parsimonious model.

To the best of our knowledge, only four simulation studies have examined the performance of (extended) mixture IRT models for polytomous items (excluding single-replication simulations), whose details are reported in Table 1. These are mixed one-, two- and three-parameter IRT models, some of which are extended by an additional class-specific parameter or random effect, allowing researchers to simultaneously unmix a sample into homogeneous latent classes and to control or quantify specific response style effects. In general, the simulation conditions of these studies included varying sample sizes (200 up to 6,000 respondents), scale lengths (4 up to 50 items), response formats (with 4 up to 6 ordered response categories), and features of latent classes (e.g., number of latent classes, class sizes). These simulation studies focused on applying mixed IRT models for the purpose of individual diagnostic and obtaining accurately estimated individuals' trait values when latent heterogeneity of a target population as well as effects of category use are taken into consideration. It is well known that IRT models require sufficiently long scales to precisely estimate individuals' trait values (Reise and Yu, 1990; DeMars, 2003; Kieftenbeld and Natesan, 2012; Meyer and Hailey, 2012; He and Wheadon, 2013). In these simulation studies the items showed only a few number of response categories to prevent estimation problems (Choi et al., 1997; De Ayala and Sava-Bolesta, 1999; French and Dodd, 1999; DeMars, 2003; De La Torre et al., 2006; Lange, 2008; He and Wheadon, 2013). Hence, all these simulation studies are characterized by (relatively) long scales (10–50 items) and few response categories (4- to 6-point rating scales).

**Table 1:** Overview of the simulation studies on the performance of mixture polytomous IRT models.

**Model description**	**Design**	**Main finding and acceptable data condition**
Huang (2016) Two mixed GPCMs with a random-effect RS-variable (the so-called mixture ERS-GPCM^a^ and the mixture ERS-GPCM-CD^b^)	Fixed factors: - Latent mixture: 3 classes [ORS class (50%), ERS class (25%), and MRS class (25%)] Varied factors: - Sample size: 200, 500, 1,000, and 2,000 cases - Scale length: 10, 15, 20, and 40 items - Rating scale: 4 and 6 categories Bayesian estimation method	Optimal performance: - *N* = 1,000 cases and 20 items. Further relevant results: - Accuracy of parameter estimates and classification rates are positively associated with longer scales, larger sample sizes, and more response options; - More accurate parameter estimates in the larger class than in small classes; - High non-convergence rate in the case of short scales and small sample sizes.
Jin and Wang (2014) The mixed 3P-GPCM with class-specific decrement parameter^c^	Fixed factors: - Sample size: 2,000 respondents - Scale length: 20 items - Rating scale: 4 categories - Unequal class sizes: 60 and 40% Varied factors: - Latent mixture: 1 and 2 classes - Decrement parameter: 0, 0.1, and 0.2 Bayesian estimation method	- Optimal parameter recovery under all simulation conditions (*RMSE* < 0.11).
Wetzel et al. (2016) The mixed PCM (mPCM; Rost, 1997)	Fixed factor: - Rating scale: 4 categories Varied factors: - Latent mixture: 1 class and 2 classes [ERS class (50%) and NERS class (50%)] - Sample size: 200, 500, and 2,000 cases - Scale length: 5, 10, 25, and 50 items Marginal maximum likelihood (MML) estimation method	- For one-class-PCM, high recovery accuracy of person parameters with 10 or more items across all sampe sizes and scale lengths. - For two-class-mPCM, the mean probabilities of class membership is high for all scale lengths; moderate accuracy of person parameters for the short scale (5 items) across all sample sizes; high accuracy of person parameters for scales with more items.
Cho (2014) The mixed PCM (mPCM; Rost, 1997) Latent classes represent RSs (ORS, ERS, MRS, or ARS).	Fixed factor: - Rating scale: 5 categories Varied factors: - Sample size: 1,200, 3,000, and 6,000 cases - Scale length: 4, 10, and 20 items - Latent mixture: 2, 3, and 4 classes - Class sizes: equal and unequal (the ORS class as a large one and each other RS class consists of 10% of the sample) Marginal maximum likelihood (MML) estimation method	Optimal performance: - For the four-class mPCM (equal classes), *N* = 3,000 cases and 10 items. Further relevant results: - The mPCM with less classes required less than *N* = 3,000 cases. - For unequal classes, more cases are needed to achieve the same accuracy compared to equal classes. - Class-specific parameters of small classes showed less accurate estimates. - The test length was the main factor affecting the accuracy of ability parameter recovery. - The test length was the most important factor for classification accuracy, regardless of the sample size. - A higher misclassification rate for small classes and classes with similar class-specific item parameters (e.g., ERS class and ARS class). Estimation problems (non-convergence and boundary values): - For the four-class mPCM (unequal classes), with a small sample size and short test length, mostly due to insufficient expected category-frequencies (near zero), especially for in small classes.

*GPCM, Generalized partial credit model; RS, response style; ORS, ordinary response style; ERS, extreme response style; MRS, middle response style; NERS, non-extreme response style; ARS, acquiescence response style, RMSE, root mean squared error; PCM, Partial credit model; mPCM, mixed partial credit model*.

a *The so-called mixture ERS-GPCM allows to detect latent classes with different response patterns and additionally quantify an individual tendency for ERS. For this purpose, it includes an additional random-effect factor that represents interindividual differences in category widths*.

b *The so-called mixture ERS-GPCM-CD is an extension of the mixture ERS-GPCM and includes an additional item specific constrained discrimination (CD) parameter. It makes possible to identify items that strongly evoke ERS*.

c *The 3P-GPCM with class-specific decrement parameter is the most complex extension of the mixed GPCM. It includes a decrement parameter which allows to quality a possible decline in respondents' response behavior (because of a time limit, low motivation or insufficient ability)*.

However, these simulation studies have hardly included the data situation that is often observed in national panel surveys and large-assessment surveys where the measurement of attitudes and traits are based on short scales [e.g., the 5-item measure of job satisfaction in the Household, Income and Labor Dynamics in Australia Survey (HILDA Survey; Summerfield et al., 2015); the 5-item measure of satisfaction with working condition in Swiss Household Panel (SHP; Voorpostel et al., 2014)]. Clearly, in context of panel studies, it is impractical to use long-scale measures, primarily to keep the time required to respond to the questionnaire within a reasonable limit to prevent any reduction in participants' motivation and to collect data of high quality. Moreover, short scales are usually compensated by a rating scale consisting of many response categories (e.g., 11-point rating scale) with the purpose to measure fine gradations of individuals' trait levels on a trait or an attitude of interest (see Krosnick and Presser, 2010; Willits et al., 2016). However, empirical research has shown that scales with many response categories are affected by reduced psychometric data quality due to increased error variance as a consequence of response styles evoked by many categories (Chang, 1994; Weng, 2004). It is precisely this data situation which makes the use of mixed IRT models particularly reasonable, enabling a researcher to explore category use patterns existing in the data and to adjust estimates of individuals' latent trait values. Thus, the present simulation study focuses on examining under which conditions (e.g., sample size) mixed IRT models for polytomous items would perform appropriately when they are applied to data assessed with a short scale (e.g., 5 items) and many response categories (e.g., 11 response categories).

### Determining the Number of Latent Classes Using Information Criteria

One critical issue in applying mixed IRT models is the determination of the number of latent classes. This is typically done by applying information criteria [e.g., Akaike's information criterion (AIC; Akaike, 1974), Bayesian information criterion (BIC; Schwarz, 1978) or consistent AIC (CAIC; Bozdogan, 1987)]. Because information criteria are differently affected by the model complexity (number of model parameters) and sample size, they usually provide inconsistent suggestions concerning the best-fitting class solution (Li et al., 2009; Cho, 2014; Yu and Park, 2014; Choi et al., 2017). Therefore, the conditions under which these information criteria perform well have to be explored. Several simulation studies have been conducted to give an answer to this question.

In her extensive simulation study, Cho (2014) examined the effectiveness of traditional information criteria such as the AIC, the BIC, and CAIC for determining the true number of latent classes of the mPCM model under different simulation conditions. She found that the BIC generally performed well in the most conditions, followed by the CAIC showing a slightly lower overall accuracy rate. In contrast, the asymptotically inconsistent AIC often overestimated the true number of latent classes, especially with larger sample sizes. Consistent findings have also been reported for mixture dichotomous IRT models (Li et al., 2009; Preinerstorfer and Formann, 2012; Cho et al., 2013). However, Cho (2014) concluded that the BIC and the CAIC are not the best. For example, both information criteria tend to underestimate the true number of latent classes in the case of an insufficient sample size (e.g., <1,000 respondents) and when complex mixture models are applied (Bozdogan, 1987; Dias, 2006; Nylund et al., 2007; Yang and Yang, 2007; Cho, 2014; Yu and Park, 2014; Choi et al., 2017). In these conditions the AIC performs better.

In other studies, the AIC whose penalty term includes the tripled number of model parameters (AIC3; Bozdogan, 1994) and the sample size adjusted BIC (SABIC; Sclove, 1987) have been proven to overperform the BIC, the CAIC, and the AIC, especially for relatively small sample sizes (Andrews and Currim, 2003; Dias, 2006; Nylund et al., 2007; Yang and Yang, 2007; Fonseca, 2010; Yu and Park, 2014; Choi et al., 2017). The AIC3 can detect the true latent mixture structure with a high accuracy rate (above 90%) almost regardless of the sample size, if it consists at least of 500 respondents (Yang and Yang, 2007; Fonseca, 2010). In contrast to the BIC, the SABIC which less penalizes the model complexity showed a lower underfitting rate under reasonably small sample sizes (Choi et al., 2017). Both the AIC3 and the SABIC were proper in detecting complex latent mixtures with more than two classes (Yang and Yang, 2007; Yu and Park, 2014). Although these simulation studies provide important insight into the appropriateness of different information criteria, it is unknown whether they behave appropriately in the context that is typical for survey research (small scales, many response categories).

### Objectives of the Study

The objective of this study is to examine the required sample size for two mixed polytomous IRT models that are primarily used for exploring category use by means of Monte Carlo simulations. The restricted mixed GPCM (rmGPCM; with varying discrimination parameters of items only for the total population but not across latent classes) and the mPCM (with equal discrimination parameters of items) are compared regarding their performance under small to large sample sizes. Both models have been well established in research on category use (e.g., Eid and Rauber, 2000; Austin et al., 2006; Meiser and Machunsky, 2008; Wetzel et al., 2013; Kutscher et al., 2017). The present simulation study primarily focuses on a realistic data situation in the field of national surveys, where psychological constructs are assessed using short scales with many response categories. To prevent the main limitation of previous simulation studies, we use empirically-based model parameters reflecting the latent mixture of three subpopulations with different category use patterns. Thus, we first examine how the rmGPCM and the mPCM with three latent classes as a true latent mixture work under varying sample sizes when applied to the challenging data (a short scale equipped with many response categories). In addition, for three sample sizes (a small, medium-sized, and a large one), we study what estimation problems can arise and how accurately the model parameters can be estimated when these models are used for different tests (e.g., long scales with a few response categories). We also include conditions of data comprising a simple latent mixture (two classes with different response styles). Furthermore, we compare different information criteria in their performance for correctly identifying the true class solution of both models. This study should provide an insight into requirements and obstacles when exploring category use by means of the mixed IRT models in the presence of a challenging data situation (5 items with 11 response categories). To the best of our knowledge, this is the first study investigating the mixed one- and two-parameter IRT model for polytomous data in the context of a short scale and a large number of response categories and, therefore, will add a valuable contribution to the literature on mixture IRT approaches.

## Materials and Methods

### Data-Generating Models

In the current simulation study, we use the rmGPCM and the mPCM (Rost, 1997) as data generating models. As a parsimonious variant of the mixed GPCM (GPCM; Muraki, 1997; mGPCM; von Davier and Yamamoto, 2004), the rmGPCM defines for each latent class the conditional probability of endorsing a response category *x* of an item *i* as a function of the latent trait variable by two types of item parameters: (i) class-specific threshold parameters that define the location of transition between two adjacent categories of an item *i* (*x* – 1 and *x*) on the latent continuum and (ii) a class-fixed discrimination parameter of an item *i* (as a multiplicative parameter) that indicates how well the item differentiates between individuals with different values on the trait that is measured. That means that the location and the order of thresholds can differ between latent classes. The discrimination parameters are freely estimated for items and are fixed across latent classes. The rmGPCM is defined by the following equation:

(1) $$\begin{array}{r} P_{vix}\left(\theta \right)=\overset{G}{\underset{g=1}{\sum }}\pi_{g}\frac{\text{exp }\left[\sum_{s=0}^{x}\delta_{i}\left(\theta_{vg}-\;\tau_{isg}\right)\right]}{\sum_{c=0}^{m}\exp\left[\sum_{s=0}^{c}\delta_{i}\left(\theta_{vg}-\tau_{isg}\right)\right]} \end{array}$$

with *x* ∈{0*,…, m*}, *s* ∈{0*,…, c*}, δ_*i*_ > 0; $\sum_{g=1}^{G}\pi_{g}$ = 1, *E*(θ_*vg*_) = 0 for all *g*, τ_*i*0*g*_ = 0 for all *i* in all *g*, δ_1_ = 1 (as identification constraints).

In Equation 1, the proportion of individuals in each latent class (class sizes) π_*g*_ (0 < π_*g*_ < 1), the class-specific threshold parameters for item *i* (τ_*isg*_), the item-specific discrimination parameters (δ_*i*_), and the class-specific values on the latent trait which are measured for person *v* (θ_*vg*_) are all model parameters to be estimated. *P*_*vix*_(θ) denotes the probability of an individual *v* endorsing a category *x* of item *i*. The number of a priori unknown subpopulations (*G*) can be determined by comparing goodness-of-fit statistics of models differing in the number of latent classes (Rost, 1997). In addition, the class membership *g* (*g* = 1,…, *G*) of each individual can be determined by his or her maximal class assignment probability. Mathematically, the mPCM (Rost, 1997) is a special variant of the rmGPCM. It assumes that the discrimination parameters do not differ between items and classes and are usually fixed to one. In both models, the threshold parameter values have the same meaning.

In the present simulation study, data generating and data analysis were implemented in the Latent GOLD 4.5 package (Vermunt and Magidson, 2008). It should be noted that in this software the parametrization of mixed IRT models is based on the generalized linear model (GLM), and, therefore, model parameters are partially generated in a different metric as commonly used in the IRT approach (e.g., difference of adjacent category parameters instead of threshold parameters). For example, the model equation for the rmGPCM has the following form of a logistic regression model:

(2) $$\begin{array}{r} log\frac{P\left(Y_{i}\;=\;m|F_{v},\;g\right)}{P\left(Y_{i}\;=\;m-1|F_{v},\;g\right)}\;=\left(\beta_{0mg}^{i}-\;\beta_{0m-1g}^{i}\right)\;+\;\lambda^{i}F_{v}, \end{array}$$

where *Y*_*i*_ is an observed response for item *i* and, *F*_*v*_ is a person's latent trait value (representing the weighted average of one's class-specific ability parameters) $\left(\beta_{0mg}^{i}-\beta_{0mg-1g}^{i}\right)$, denotes a parameter for the difference of category difficulty parameters of two adjacent categories *m* and *m* – 1 for item *i* in the class *g* (the so-called delta beta parameter, ${\Delta \beta }_{0sg}^{i}$), and λ^*i*^ is an item discrimination parameter. The results are reported with respect to the Latent GOLD parameterization.

### Simulation Design

The present simulation study primarily examined what sample size is required to avoid estimation problems and to obtain accurately estimated model parameters, standard errors and correct model fit coefficients for the challenging data characterized by a short scale and a large number of response categories (namely, a 5-item scale with 11 response categories).

To strengthen the ecological validity of the simulation study, the data-generating models used under this data condition were the three-class rmGPCM and the three-class mPCM (described in their general form in the previous section). The generating parameter values of both models (taken as population parameters) were drawn from an application to empirical survey data reported by Kutscher et al. (2017) and are shown in Table 2. In this empirical application, five items measuring job satisfaction on an 11-point rating scale from the first wave of the Household, Income and Labor Dynamics in Australia [HILDA] Survey (Summerfield et al., 2015) were analyzed. Fitting the data with both models, three latent classes with different category use were detected based on a subsample of 7,036 employees and employers. In this application, the three-class rmGPCM showed the best-fit. The three classes can be characterized as follows: The first class shows an ERS with a large number of avoided categories (indicated by many unordered thresholds); the second class is characterized by a roughly ordinary response style (ORS) and a few avoided response categories (indicated by approximately equal widths between adjacent threshold parameters and a few unordered thresholds); members of the third class prefer the two lowest and two highest response categories (semi-ERS) with many avoided categories between. Therefore, the ORS class and the ERS class substantially differ in their class-specific item parameters, while the semi-ERS class has a certain similarity to each of these latent classes. In that application, the class sizes were as follows: 0.33, 0.40, and 0.27 for the rmGPCM and 0.32, 0.43, and 0.25 for the mPCM. Notably, these class sizes are consistent with previous findings, suggesting that most respondents usually show an appropriate category use and a third of a sample prefers the ERS (cf. Eid and Rauber, 2000; Wetzel et al., 2013).

**Table 2:** Generating parameters of the rmGPCM-3 (upper lines) and the mPCM-3 (bottom lines).

	**λ^*i*^**	$\Delta \beta_{01\text{g}}^{i}$	$\Delta \beta_{02\text{g}}^{i}$	$\Delta \beta_{03\text{g}}^{i}$	$\Delta \beta_{04\text{g}}^{i}$	$\Delta \beta_{05\text{g}}^{i}$	$\Delta \beta_{06\text{g}}^{i}$	$\Delta \beta_{07\text{g}}^{i}$	$\Delta \beta_{08\text{g}}^{i}$	$\Delta \beta_{09\text{g}}^{i}$	$\Delta \beta_{010\text{g}}^{i}$	**λ_*g*_**	**π_*g*_**
**Class 1**													
Item 1	1^a^	*−0.98*	0.77	*0.52*	0.08	*1.22*	−0.61	0.78	0.24	*−1.24*	*1.89*		
	1	*−0.87*	0.85	*0.58*	0.16	*1.25*	−0.58	0.80	0.24	*−1.26*	*1.86*		
Item 2	0.71	*−0.79*	0.71	*−0.07*	0.03	*1.26*	−1.04	0.56	0.96	*−0.69*	*2.49*		
	1	*−0.59*	0.94	*0.09*	0.20	*1.38*	−0.94	0.64	1.02	*−0.70*	*2.43*		
Item 3	1.27	*−0.28*	1.07	*0.56*	0.07	*1.76*	−0.32	0.81	0.62	*−0.94*	*2.05*	0.21	0^a^
	1	*−0.20*	0.98	*0.59*	0.09	*1.76*	−0.29	0.80	0.64	*−0.97*	*2.08*	0.28	0^a^
Item 4	2.57	*−0.31*	1.98	*0.66*	0.39	*1.97*	−0.55	0.88	0.75	*−1.43*	*2.27*		
	1	*−0.74*	1.39	*0.23*	0.00	*1.72*	−0.81	0.76	0.72	*−1.39*	*2.45*		
Item 5	1.76	*−0.52*	1.00	*0.32*	0.28	*1.63*	−1.12	1.21	0.67	*−0.95*	*2.43*		
	1	*−0.67*	0.73	*0.18*	0.18	*1.53*	−1.27	1.21	0.67	*−0.95*	*2.54*		
**Class 2**													
Item 1	1^a^	*2.27*	1.52	*0.86*	0.40	0.64	*0.25*	0.46	*0.10*	*−1.92*	−1.16		
	1	*2.01*	1.39	*0.94*	0.42	0.74	*0.20*	0.48	*0.06*	*−1.66*	−1.32		
Item 2	0.71	*1.72*	1.21	*0.83*	*−0.03*	0.82	*0.04*	0.59	0.70	*−0.67*	−0.21		
	1	*1.96*	1.34	*1.05*	*0.11*	0.98	*0.06*	0.70	0.67	*−0.55*	−0.25		
Item 3	1.27	−3.10	*4.00*^b^	*1.80*	*0.58*	1.05	*0.38*	0.68	0.45	*−1.38*	−1.20	0.24	0.20^c^
	1	0.63	*2.78*	*1.58*	*0.54*	1.02	*0.34*	0.68	0.42	*−1.20*	−1.01	0.30	0.30^c^
Item 4	2.57	*4.00*^b^	3.28	*1.70*	1.07	1.05	*0.28*	0.59	*0.06*	*−2.21*	−1.73		
	1	4.00^b^	*2.02*	*1.10*	0.5*5*	0.76	*0.03*	0.51	*0.1*2	*−2.12*	−1.86		
Item 5	1.76	*1.91*	1.86	*0.98*	*0.38*	0.84	*0.30*	0.59	0.29	*−0.99*	−0.93		
	1	*1.49*	1.43	*0.73*	*0.19*	0.68	*0.15*	0.55	0.27	*−0.96*	−0.98		
**Class 3**													
Item 1	1^a^	*1.61*	0.60	*0.35*	*−0.22*	0.83	0.01	0.85	0.03	*0.11*	*−1.93*		
	1	*1.73*	0.78	*0.40*	*−0.15*	0.80	0.17	0.84	0.04	*0.01*	*−1.89*		
Item 2	0.71	*0.27*	0.20	*−0.43*	0.48	1.01	*−0.94*	1.58	0.23	*0.96*	*−0.66*		
	1	*0.47*	0.44	*−0.35*	0.71	1.07	*−0.69*	1.46	0.27	*0.86*	*−0.74*		
Item 3	1.27	*2.27*	0.60	*0.34*	*−0.01*	1.06	0.33	0.87	0.20	*0.96*	*−1.46*	0.21	−0.18
	1	*2.27*	0.59	*0.29*	*−0.13*	1.26	0.37	0.91	0.29	*0.93*	*−1.53*	0.28	−0.25
Item 4	2.57	*4.00*^b^	0.65	*0.79*	0.22	1.51	*0.21*	0.63	0.12	*0.77*	*−2.03*		
	1	*4.17*	0.13	*0.16*	*−0.34*	1.56	*0.1*8	0.65	0.33	*0.95*	*−1.68*		
Item 5	1.76	*1.15*	0.68	*0.26*	0.32	1.04	*−0.32*	0.63	0.54	*0.78*	*−0.70*		
	1	*0.90*	0.51	*−0.13*	0.29	1.27	*−0.37*	0.63	0.68	*0.94*	*−0.56*		

*λ^i^, discrimination parameter; ${\Delta \beta }_{0sg}^{i}=\left(\beta_{0mg}^{i}-\;\beta_{0m-1g}^{i}\right)$, delta beta parameter; λ_g_, estimate of the variance of the class-specific latent trait variable; π_g_, latent class-size parameter. All parameters are given in Latent GOLD metric (see Vermunt and Magidson, 2006). The delta beta parameters used as the population parameters under the conditions of tests with six response categories are given in italics*.

a *Default setting*.

b *Extreme parameters were substituted by |4|*.

c *For the population models with a two-class mixture, the class-size parameter of the second latent class is set to 0.75*.

For this data condition (namely, a short test with many response categories and three-class mixture), two factors were manipulated in the simulation study: (i) model type (the rmGPCM, the mPCM) and (ii) sample size (starting from 500 observations up to 5,000 observations with a step of 500 observations). Sample sizes were chosen to represent realistic data conditions. These two manipulated factors were crossed, resulting in 20 simulation conditions. Five hundred replications were generated per sample size condition. Within a replication, we estimated the one- to four-class solutions of a corresponding model.

To increase the generalizability of the results from the present simulation study, we included three additional data conditions: a large scale with many response categories (15 items with 11 categories), a short scale with a few response categories (5 items with 6 categories), and a typically designed test (15 items with 6 categories). Thus, these data conditions should provide evidence of how the performance of two mixed IRT models may improve by increasing the number of items, reducing the number of response categories, or by using a traditionally designed test. In doing so, to generate the responses for the 6-category conditions, 5 of 10 delta beta parameters of each item were selected from the empirical application of the rmGPCM and the mPCM (see in Table 2 for item parameters that are given in italics). The selected item parameters represented the three-class mixture with the identical response styles, as described for the challenging data condition. To create 15-item tests, the item parameters of the five items were taken triple. In addition, we have also examined how the rmGPCM and the mPCM would work under four different data conditions (incl. a 5-item test with 11 response categories) when the data comprises a simple latent mixture of two classes. This type of latent mixture has often been found in empirical studies (for example, see Wetzel et al., 2013). To generate responses under the two-class mixture, the model parameters of the first two classes obtained from an empirical application of the rmGPCM and the mPCM, as described above, were used. We only optimized the class-size parameters so that the first class (ERS class) included approximately a third of a sample (33%) and the second class (ORS class) contained two-thirds of cases (67%). Finally, we varied sample size (*N* = 1,000, 2,500, and 4,500) within each of additional conditions. These sample sizes represent a small, medium-sized, and a large sample, respectively. This resulted in 42 conditions. Fifty replications were generated per condition. Within each replication, we estimated the two- to four-class solutions when the population model represents a three-class mixture and the one- to three-class solutions in the case of a true two-class mixture.

In the present study, the marginal maximum likelihood (MML) estimator implemented in Latent GOLD was used for estimation of both models. For effective MML estimation, the stable EM algorithm (Bock and Aitkin, 1981) is used in the initial stage of the estimation process and it switches to the speedy Newton-Raphson (NR) method in the final stage (Vermunt and Magidson, 2013). Each estimation algorithm stops when its maximum number of iterations or the convergence criterion (equals to 0.01 and 10^−8^ per default for the EM algorithm and the NR algorithm, respectively) is reached. In order to prevent estimation problems (such as non-convergence or local maximum), the following estimation options were chosen, for all class solutions in all sample size conditions: (i) the number of iterations for the EM algorithm and the NR method were fixed to 10,000 and 600, respectively; (ii) the number of multiple sets of starting values was set to 100 and the number of EM iterations performed within each start set was set to 200; (iii) following Muraki suggestion (1997), the number of quadrature points was set to 80. Further options were left to Latent GOLD default values.

### Analyses

#### Monitoring Convergence and Estimation Problems

To evaluate the estimation performance of the rmGPCM and the mPCM, convergence checks were conducted for each analysis of each replication by considering the convergence rate of the EM algorithm and the NR estimation method and the occurrence of boundary values. Latent GOLD indicates these estimation problems with warning messages. Consequently, replications with warning messages were inspected. A high rate of boundary values within a class solution (e.g., over 10%) is indicative for an improper solution. In the conditions of a true three-class mixture, replications with an improper three-class solution of rmGPCM and mPCM were eliminated from sequential analyses (on details on this issue, see Results section). We proceeded identically in the conditions of a true two-class mixture.

#### Detection of Label Switching

Evaluating the estimation accuracy of the rmGPCM and the mPCM across replications requires the match in the order of latent classes between the data-generating model and replications (exclusion of label switching). A useful approach to detect switched classes within a replication is based on comparing class-specific item parameters used for data generating with the estimates from each replication (see Li et al., 2009; Cho et al., 2013; Cho, 2014).

In the present simulation study, label switching should actually be prevented by using data-generating parameters as starting values for estimating three-class rmGPCM and the three-class mPCM in the corresponding replications within the three-class mixture conditions (Vermunt and Magidson, 2016). The same holds for the conditions of the two-class mixture. To ensure that it had worked well, we checked the occurrence of switched classes by means of the multinomial logistic regression analysis within each condition. This method was based on variables containing delta beta parameter estimates from replications and predicted their assignment to a certain latent class. For example, 50 variables of delta beta parameters were used in the condition of a 5-item scale with 11 categories. In all conditions, perfect correspondence between observed and predicted class assignments of delta beta parameters was found (complete separation). Hence, as expected, no label switching occurred.

#### Measures of Estimation Accuracy

The estimation accuracy was evaluated using the following robust accuracy indices: Root median squared error (*RMdSE*), standard error bias (*bias*_*s*e_), median width of the confidence interval (*Md*_*width*_*CI*__), Spearman's rank correlation coefficient (*r*_*s*_), and 95% coverage. We primarily used the median-based measures that are robust to extreme estimates that might occur as a consequence of the sparse data problem. The preliminary analysis revealed that the estimates of a particular parameter or standard error across replications were approximately normally distributed. However, the estimates of lower thresholds primarily indicated some large values on one side of the distribution. This would make the results obtained using mean-based indices (e.g., RMSE) questionable.

The *RMdSE* is a robust measure of absolute accuracy of parameter estimation computed by

(3) $$\begin{array}{r} RMdSE=\sqrt{Md_{{\left(\hat{p}-p\right)}^{2}}} \end{array}$$

where $\hat{p}$ denotes the parameter estimate of the *t*th replication and *p* represents the generating parameter value. Thus, this index is based on squared differences between estimated and the true parameters each of those is calculated for a replication; the squared root of median is then used to aggregate these differences across replications. The less parameter estimates across replications deviate from the true parameter value, the smaller the *RMdSE* is observed.

The standard error bias (*bias*_*se*_) demonstrates how well the standard error of a parameter is reproduced by the standard deviation of empirical distribution of its estimates across replications. Thus, standard error bias was calculated as the median of absolute differences between standard error ($\hat{se}$) estimate of a parameter in *t*th replication and empirical standard deviation ($SD_{\hat{p}}$) of parameter estimates across all replications:

(4) $$\begin{array}{r} bias_{se}=Md_{\left|\hat{s}e\;-SD_{\hat{p}}\right|} \end{array}$$

If standard error estimates of a parameter are close to the empirical standard deviation of the parameter distribution, the *bias*_*se*_ should be close to zero.

The median width of confidence interval (*Md*_*width*_*CI*__) is a robust measure of the estimation accuracy of standard errors. Small standard errors affect narrow confidence intervals and thus indicate accurate parameter estimation. For a parameter in the *t*th replication, the width of 95% confidence interval was calculated using the estimated standard error and the 97.5th quantile of the standard normal distribution; then, the median was used to aggregate these statistics across replications as follows:

(5) $$\begin{array}{r} Md_{{width}_{CI}}{=Md}_{\left({2^{*}z}_{{\left(.975\right)}^{*}}\hat{se}\right)} \end{array}$$

To obtain only one statistic for the *RMdSE, bias*_*se*_, and *Md*_*width*_*CI*__ across delta beta parameters in latent classes, all calculated accuracy indices were aggregated based on the median. Similarly, the average coverage was calculated, separately for latent classes. Before calculating the estimation accuracy indices, extreme parameter estimates (>|10|), extreme standard error estimates (>50), and boundary values of standard errors, including their corresponding standard errors and parameters, were eliminated (for details, see Result section). The cutoff values for extreme parameter and standard error estimates were set based on their empirical distributions found across the replications.

In addition, for class-specific delta beta parameters of each item, Spearman's rank correlation (*r*_*s*_) was calculated between the population parameters and their estimates within a replication. It provides how accurately these estimates represent a class-specific response pattern which is inherent in the data-generating parameters. Correlation coefficients were then averaged across replications and items. A high average correlation coefficient (at least 0.90) demonstrates the highly concordant order of estimated and generating delta beta parameters in latent classes.

Finally, the 95% coverage was calculated that reflects the proportion of replications for which a 95% confidence interval covers the generating parameter value. We considered the coverage rate of at least 0.90 to be optimal. All analyses were performed using R 3.3.0 (R Core Team, 2016).

#### Detection of the True Class Solution

The current study evaluated how effective five information criteria implemented in Latent GOLD are for identifying a true class solution for applications of the rmGPCM and the mPCM under the different data conditions and latent mixtures. The information criteria considered are defined as follows:

(6) $$\begin{array}{r} \text{AIC}=-2LL+2^{*}N_{par} \end{array}$$

(7) $$\begin{array}{r} \text{BIC}=-2LL+log{\left(N\right)}^{*}N_{par} \end{array}$$

(8) $$\begin{array}{r} \text{CAIC}=-2LL+{\left[log\left(N\right)+1\right]}^{*}N_{par} \end{array}$$

(9) $$\begin{array}{r} \text{AIC3}=-2LL+3^{*}N_{par} \end{array}$$

(10) $$\begin{array}{r} \text{SABIC}=-2LL+[log\left(\frac{\text{N}+2}{24}\right){]}^{*}N_{par} \end{array}$$

where −2*LL* is −2 times the log-likelihood of the class solution, *N*_*par*_ is the number of parameters to be estimated, and *N* is the sample size.

The class solution with the smallest value of an information criterion is indicated as the best-fitting model. In the present study, for each information criterion coefficient, the proportion of replications in which a specific class solution of the rmGPCM or the mPCM was identified as the best-fitting model was calculated and compared under different sample size conditions. We considered an information criterion as appropriate when it could correctly identify the true class solution at least in 95% of replications generated by the corresponding mixed IRT model.

## Results

### Convergence and Estimation Problems Under the Challenging Data Condition

Table 3 gives an overview of convergence and estimation problems for the three-class rmGPCM (rmGPCM-3) and the three-class mPCM (mPCM-3) when the data comprised a true three-class mixture. For the rmGPCM-3, the EM algorithm converged in all replications. Contrarily, the convergence rate of the rapid NR algorithm reached only 69% of replications across all sample size conditions and this is considerably reduced with increasing sample size (from 84 to 56% with *N* = 500 and *N* = 5,000, respectively). Coincidently, boundary estimates occurred in almost all non-convergent replications. A detailed analysis revealed that the boundary values problem mostly referred to the standard error estimates of the same delta beta parameters ${\Delta \beta }_{02,\;g=2}^{i=3}$ (in 83% of non-convergent replications). Note that in the empirical application of the rmGPCM-3 to HILDA data (population model), this parameter was estimated to be extreme (see Table 2) because the expected frequencies of two lower categories of item 3 in the second class were null (see Table S7). Obviously, a sparse table seems to be a challenge for the NR method. By increasing the sample size, the high rate of boundary estimates of the standard error concerned (and consequently that of non-convergent replications) may be explained by the fact that these adjacent response categories still did not provide sufficient data points required for accurate estimation of this delta beta parameter by the NR algorithm in the certain sample size condition. Furthermore, seven replications across all conditions were identified as improper (see in parentheses in the column “BV_SE_” in Table 3) and were completely excluded from the subsequent analyses.

**Table 3:** Convergence rates of the EM algorithm and the Newton-Raphson algorithm, number of required iterations, occurrence of boundary values and improper solution, and mean classification probability for the rmGPCM-3 and the mPCM-3 under the condition of a true three-class mixture and a 5-item scale with 11 categories.

***N***	**Conv. EM, %**	***Md*_EM_ (Range_EM_)**	**Conv. NR, %**	***Md*_NR_ (Range_NR_)**	**BV_SE_, % (improper)**	***M*_P(Y|G)_**
**rmGPCM3**						
500	100	303 (111–2,133)	84	8 (5–600)	16 (3)	0.88
1,000	100	256 (99–1,256)	79	8 (4–600)	21 (1)	0.85
1,500	100	197 (67–1,467)	69	9 (3–600)	31 (0)	0.84
2,000	100	160 (58–1,158)	68	9 (3–600)	32 (0)	0.83
2,500	100	139 (59–956)	72	9 (3–600)	28 (1)	0.82
3,000	100	127 (54–402)	68	9 (3–600)	32 (1)	0.82
3,500	100	122 (58–1,897)	65	9 (3–600)	35 (0)	0.82
4,000	100	110 (49–377)	62	9 (3–600)	38 (0)	0.82
4,500	100	109 (51–373)	62	9 (3–600)	38 (0)	0.82
5,000	100	99 (50–603)	56	10 (3–600)	44 (1)	0.82
**mPCM3**						
500	100	293 (126–1,188)	96	8 (6–600)	4.4 (0)	0.89
1,000	100	247 (75–1,415)	98	8 (4–600)	2.2 (0)	0.86
1,500	100	208 (70–1,033)	99	8 (3–600)	0.8 (0)	0.85
2,000	100	171 (66–920)	99.6	8 (3–600)	0.4 (0)	0.84
2,500	100	151 (50–719)	99.8	7 (3–600)	0.2 (0)	0.84
3,000	100	138 (60–677)	100	7 (2–23)	0	0.84
3,500	100	124 (57–497)	100	6 (2–19)	0	0.83
4,000	100	119 (53–506)	100	6 (2–21)	0	0.83
4,500	100	112 (47–541)	100	6 (2–14)	0	0.83
5,000	100	110 (55–331)	100	5 (3–17)	0	0.83

*N, sample size condition; Conv.EM, convergence rate of the EM algorithm; Md_EM_ (Range_EM_), median (range) of iterations required to reach a convergent solution of the EM algorithm; Conv.NR, the convergence rate of the Newton-Rapson algorithm; Md_NR_ (Range_NR_), median (range) of iterations required to reach a convergent solution of the Newton-Rapson algorithm (Note, solutions with 600 iterations did not converge); BV_SE_ (improper), proportion of replications with boundary values (the number of replications with an improper solution); M_P(Y|G)_, mean classification probability*.

In turn, the mPCM-3 showed more satisfactory results (see the bottom part of Table 3). The EM algorithm also converged in all sample size conditions. The non-convergence rate of the NR algorithm was maximal 4% and concerned only sample size conditions with <3,000 cases. This was mostly combined with the occurrence of the standard error of (extreme) delta beta parameters indicating a boundary value. No improper solutions were found. Regarding other class solutions of the two models, the same non-convergence and estimation problems in a greater extent were found for the four-class solutions (see Tables S1, S2). To conclude, the NR algorithm can fail to achieve a convergent solution in a case of a high model complexity (e.g., mixed multi-parameter IRT model, many latent classes) and in the presence of sparse data.

### Accuracy of Estimates Under the Challenging Data Condition

At first, we examined the amount of extreme values of the parameter and standard error estimates in the three-class solutions. For the rmGPCM-3, a total of 3% of the parameters and a few standard errors (0.03%, excl. boundary values) were estimated to be extreme in all replications across all sample size conditions. Mostly, it referred to four delta beta parameters (${\Delta \beta }_{01,g=3}^{i=4}$, ${\Delta \beta }_{02,g=2}^{i=4}$, ${\Delta \beta }_{01,g=2}^{i=3}$, ${\Delta \beta }_{01,g=2}^{i=4}$) whose values in the population model are also relatively high (see Table 2). The standard errors of the first and second delta beta parameters often obtained extreme values, primarily because of the sparse table problem concerning the lower response categories (see Table S7). For the same reasons, the mPCM-3 produced a few extreme values of parameter estimates (0.01%) and of standard errors (0.4%) across all replications. All extreme estimates, boundary values, and their corresponding parameters and standard errors were excluded from the following analysis. Below, we will report relevant results separately for accuracy indices. (Details on the distributions of accuracy indices under the different sample size conditions are provided in Tables S3–S6).

#### Root Median Standard Error

**Figure 1:**
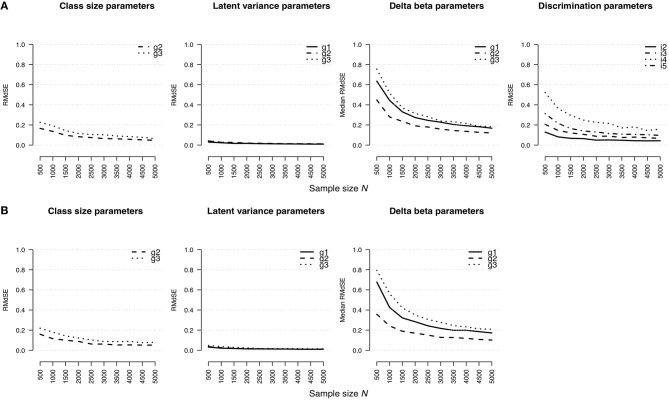
Root median squared error for parameter estimates in **(A)** the rmGPCM-3 and **(B)** the mPCM-3 under the condition of a three-class mixture and a 5-item scale with 11 categories.

Figure 1 shows an effect of the sample size on the estimation bias of parameter types regarding the rmGPCM-3 (Figure 1A) and the mPCM-3 (Figure 1B). For both models, the *RMdSE* values generally decreased by increasing the sample size with the exception of the class-specific variances of the latent trait variable that were accurately estimated already with the smallest sample size (maximal *RMdSE*^*N* = 500^ = 0.04 and 0.05 for the rmGPCM-3 and the mPCM-3, respectively). Furthermore, the class-specific class-size parameters were also only slightly biased (maximal *RMdSE*^*N* = 500^ = 0.23 and 0.22 and maximal *RMdSE*^*N* = 5, 000^ = 0.06 and 0.08 for the rmGPCM-3 and the mPCM-3, respectively). In contrast, the estimation bias was higher for both types of item parameters across all sample size conditions (for class-specific delta beta parameters, maximal *RMdSE*^*N* = 500^ = 0.76 and 0.80 and maximal *RMdSE*^*N* = 5, 000^ = 0.18 and 0.21 for the rmGPCM-3 and the mPCM-3, respectively; for item discrimination parameters of the rmGPCM-3, maximal *RMdSE*^*N* = 500^ = 0.52 and maximal *RMdSE*^*N* = 5, 000^ = 0.16). For both types of item parameters, the *RMdSE* curves show an inflection point at *N* = 1,500 indicating a sufficient decline in bias up to this sample size while further increasing the sample size had only a slight effect on the reduction of the *RMdSE* values. Discrimination parameters of items possessing higher discrimination power were estimated less accurately (e.g., item 4). Furthermore, the class size additionally affected the amount of bias for the class-specific parameters (e.g., class sizes and delta beta parameters). In particular, the parameters of the largest class (g2) were estimated more accurately compared to those of the smaller classes (g1 and g3).

#### Standard Error Bias

**Figure 2:**
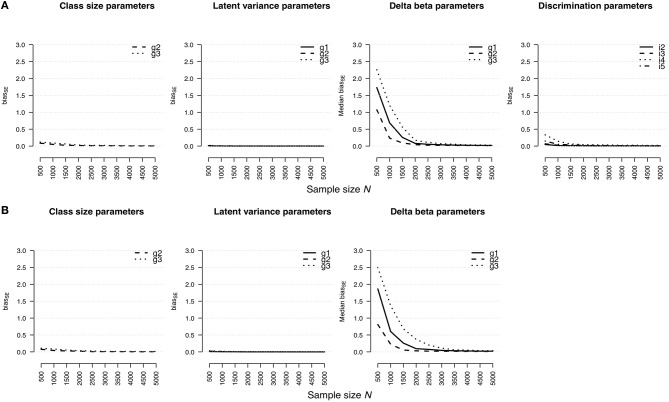
Bias of standard error estimates for parameter estimates in **(A)** the rmGPCM-3 and **(B)** the mPCM-3 under the condition of a three-class mixture and a 5-item scale with 11 categories.

The accuracy of standard error estimates for the rmGPCM-3 and the mPCM-3 is illustrated in Figures 2A,B, respectively. In general, the results are mostly identical with those reported for the *RMdSE*. A slight bias was found for the standard errors of latent variances (maximal $bias_{se}^{N=500}$ = 0.02 and 0.03 for the rmGPCM-3 and the mPCM-3, respectively) and the class-size parameters (maximal $bias_{se}^{N=500}$ = 0.12 and 0.11 for the rmGPCM-3 and the mPCM-3, respectively). In turn, the standard error estimates of item parameters were more biased in the case of small sample sizes but they showed a rapid reduction of bias by increasing the sample size: The bias was below 0.10 from *N* = 1,500 on for standard error estimates of the discrimination parameters and from *N* = 2,000 on for those of the delta beta parameters of both models. Exceptionally, the standard error bias of delta beta parameters of the small class (g3) could be accurately estimated from *N* = 2,500 and *N* = 3,000 on for the rmGPCM-3 and the mPCM-3, respectively. In addition, item discrimination size and class sizes had an additional effect on standard error bias values.

#### Median Width of Confidence Interval

**Figure 3:**
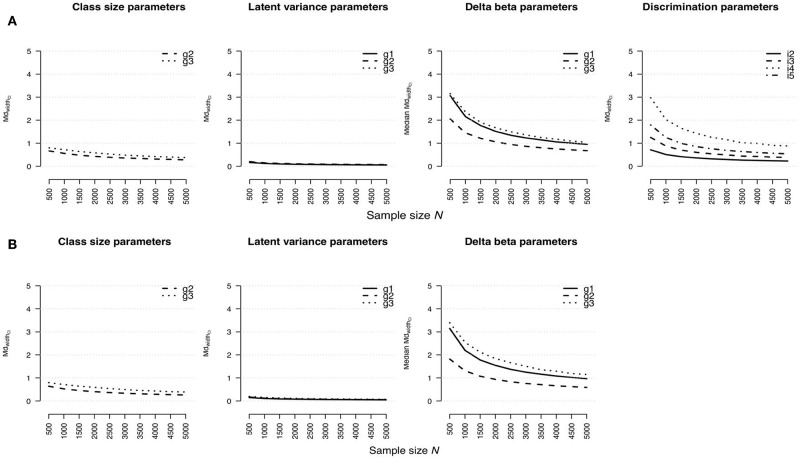
Width of confidence interval for parameter estimates in **(A)** the rmGPCM-3 and **(B)** the mPCM-3 under the condition of a three-class mixture and a 5-item scale with 11 categories.

Similar tendencies were also found for the widths of confidence intervals for model parameter estimates (see Figures 3A,B for the rmGPCM-3 and the mPCM-3, respectively). Small standard errors and consequently narrow confidence intervals were estimated primarily for both the latent variances (maximal $Md_{{width}_{CI}}^{N=500}$ = 0.20 for both models) and class sizes (maximal $Md_{{width}_{CI}}^{N=500}$ = 0.80 for both models) even with a small sample size. Again, confidence intervals of item parameters were comparably wider (for delta beta parameters, maximal $Md_{{width}_{CI}}^{N=500}$ = 3.15 and 3.40 and maximal $Md_{{width}_{CI}}^{N=5,000}$ = 1.04 and 1.15 for the rmGPCM-3 and the mPCM-3, respectively; for discrimination parameters, maximal $Md_{{width}_{CI}}^{N=500}$ = 2.97 and maximal $Md_{{width}_{CI}}^{N=5,000}$ = 0.88 for the rmGPCM-3). For these parameters, the inflection point was observed at *N* = 1,500 with small confidence intervals from that point on. Identically, larger standard errors and consequently wider confidence intervals were also found for class-specific parameters of smaller classes and large discrimination parameters.

#### Estimation Accuracy for Specific Delta Beta Parameters

All three accuracy indices pointed out that, especially the delta beta parameters and their standard errors of both models were more biased compared to other parameter types. In particular, it concerned the first five delta beta parameters (see Figures S1–S6 for the rmGPCM-3 and the mPCM-3, respectively). The first delta beta parameter in the ORS class (g2) showed high accuracy indices even with the largest sample size. Primarily, it may be caused by the low frequency of the lower categories expected for all classes, but especially for the ORS class (see Tables S7, S8 for the rmGPCM-3 and the mPCM-3, respectively). By contrast, the upper five delta beta parameters were estimated more accurately already with the medium-sized samples. Furthermore, the amount of bias of the delta beta parameters is linked to the response style. The accuracy indices were smaller in the ERS and semi-ERS classes (g1 and g3, respectively) for the lower and the upper delta beta parameters and in ORS class for the middle ones.

#### Spearman's Rank Correlation

Table 4 reports averaged Spearman's rank correlations between generating and estimated delta beta parameters. In general, for both models, the correlation coefficients in latent classes increased with enlarging the sample size, indicating that the order of estimated parameters is more and more in accordance with that of the generating parameters. Delta beta parameters of the rmGPCM-3 showed a high concordance in order (above *r*_*s*_ = 0.90) with at least *N* = 1,500 cases for two first classes (ERS class and ORS class). For the small class (semi-ERS class), a larger sample size was needed (at least *N* = 3,500), primarily because the delta beta parameters of this class were generally less accurately estimated (as reported above). For the mPCM-3, we found very similar results (see right column of Table 4).

**Table 4:** Averaged Spearman's rank correlations between the generating and estimated ${\Delta \beta }_{0sg}^{i}$-parameters for the rmGPCM-3 and the mPCM-3 under the condition of a true three-class mixture and a 5-item scale with 11 categories.

	**rmGPCM-3**			**mPCM-3**		
***N***	**g1**	**g2**	**g3**	**g1**	**g2**	**g3**
500	0.77	0.70	0.59	0.76	0.75	0.58
1,000	0.88	0.85	0.71	0.88	0.87	0.70
1,500	0.93	0.91	0.78	0.94	0.93	0.73
2,000	0.95	0.95	0.83	0.96	0.96	0.81
2,500	0.97	0.97	0.87	0.98	0.98	0.85
3,000	0.98	0.98	0.89	0.99	0.99	0.87
3,500	0.99	0.99	0.92	1.00	0.99	0.90
4,000	0.99	1.00	0.92	1.00	0.99	0.90
4,500	1.00	1.00	0.93	1.00	1.00	0.92
5,000	1.00	1.00	0.93	1.00	1.00	0.92

*g1, g2, and g3 indicate three latent classes of two models*.

#### Coverage

Table 5 reports the coverage values for parameter types of the two generating models. In general, class-size parameters showed good coverage (≥0.90) from the medium-sized samples (*N* = 2,500) on. The class-specific variances of the latent trait demonstrated good coverage rate (≥0.90) even for the relatively small sample for the rmGPCM-3 (from *N* = 1,000 on) and with medium-sized samples for the mPCM-3 (from *N* = 2,500 on). In the case of small samples, the insufficient coverage of these parameter types can be explained by too narrow confidence intervals resp. small standard errors. In turn, item parameters generally achieved acceptably high coverage in all sample size conditions (above 0.94 for discrimination parameters and above 0.93 and 0.94 for delta beta parameters of the rmGPCM-3 and the mPCM-3, respectively).

**Table 5:** Coverage for parameters of the rmGPCM-3 and the mPCM-3 under the condition of a true three-class mixture and a 5-item scale with 11 categories.

	***N***										
**Parameter type**	**Class**	**500**	**1,000**	**1,500**	**2,000**	**2,500**	**3,000**	**3,500**	**4,000**	**4,500**	**5,000**
**rmGPCM-3**											
π_*g*_	2	**0.82**	**0.85**	0.91	0.91	0.92	0.93	0.93	0.94	0.94	0.95
	3	**0.80**	**0.81**	**0.84**	**0.89**	0.92	0.90	0.92	0.92	0.93	0.93
λ_*g*_	1	0.92	0.92	0.94	0.95	0.94	0.94	0.94	0.92	0.92	0.95
	2	**0.88**	0.91	0.91	0.93	0.94	0.94	0.96	0.93	0.94	0.92
	3	0.92	0.90	0.93	0.90	0.93	0.91	0.95	0.91	0.93	0.90
${\Delta \beta }_{0sg}^{i}$ ^a^	1	0.96	0.96	0.96	0.96	0.96	0.95	0.96	0.95	0.96	0.95
	2	0.94	0.93	0.94	0.94	0.94	0.94	0.94	0.94	0.94	0.94
	3	0.95	0.95	0.96	0.96	0.96	0.96	0.95	0.96	0.95	0.95
λ^i*a*^		0.95	0.96	0.96	0.96	0.95	0.95	0.95	0.94	0.94	0.94
**mPCM-3**											
π_*g*_	2	**0.82**	**0.85**	**0.88**	0.90	0.94	0.92	0.91	0.95	0.95	0.93
	3	**0.78**	**0.83**	**0.85**	**0.86**	0.91	0.92	0.92	0.91	0.90	0.92
λ_*g*_	1	**0.88**	0.91	0.92	0.93	0.93	0.91	0.94	0.92	0.94	0.90
	2	**0.87**	**0.89**	0.91	0.93	0.94	0.95	0.94	0.94	0.95	0.95
	3	**0.84**	**0.89**	**0.88**	**0.88**	0.92	0.94	0.95	0.91	0.94	0.91
${\Delta \beta }_{0sg}^{i}$ ^a^	1	0.96	0.96	0.96	0.96	0.96	0.95	0.96	0.95	0.96	0.95
	2	0.94	0.94	0.94	0.95	0.94	0.94	0.95	0.95	0.95	0.95
	3	0.95	0.96	0.96	0.96	0.96	0.96	0.96	0.96	0.96	0.96

*π_g_, latent class-size parameter; λ_g_, estimate of the variance of the class-specific latent trait variable; ${\Delta \beta }_{0sg}^{i}$, delta beta parameter; λ^i^, item discrimination parameter*.

a *Mean coverage is reported for this parameter type*.

*Coverage rate under 0.90 is shown in bold*.

To conclude, under the challenging data condition, the two complex mixture models (with a true three-class mixture) mostly showed similar trends of estimation accuracy with varying sample size. Primarily, an accurate estimation of item parameters and their standard errors generally requires a larger sample size (at least 1,500–2,000 observations) than the other parameter types. On the contrary, the class-size parameters and the variances of a latent trait could reach a high coverage rate with at least 2,500 observations. Beyond the sample size, both the size of the latent classes and the expected category frequencies are further influential factors for estimation accuracy.

### Performance of the Mixture IRT Models Under Other Data Conditions

#### Combined With a True Three-Class Mixture

When the data reflected a three-class mixture, the rmGPCM-3 and mPCM-3 showed 100% convergence of the EM algorithm and the NR method when applied to the data characterized by a long test with many response categories, a short test with few response categories, or a long test with few categories. None of the replications indicated boundary values and no improper solutions were found (for details, see Table S9).

**Figure 4:**
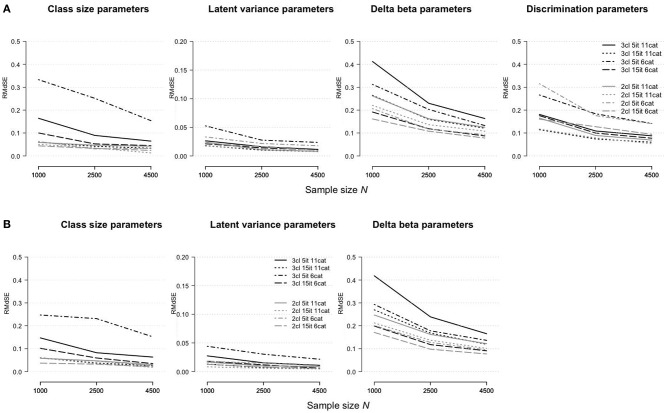
Root median squared error for parameter estimates in **(A)** the rmGPCM-3 and **(B)** the mPCM-3 under further data conditions.

Figure 4 shows how the parameter estimation bias (assessed as the *RMdSE*) differs for the rmGPCM-3 (part A) and mPCM-3 (part B) under different test and sample size conditions. Both models indicated similar tendencies: (i) a reduction of bias by increasing the sample size, regardless of the type of test, and (ii) a higher estimation accuracy of the class-size parameters and latent variances than for the item parameters. Compared to the challenging data condition as the reference condition (drawn as a solid black line), all parameter types could be estimated more precisely with long tests, suggesting that enlarging test length increases the accuracy of estimates. Conversely, in the condition of a short test with a few categories, a larger bias was observed for the class-size parameters (*RMdSE*^*N* = 1, 000^ = 0.33 and 0.25 and *RMdSE*^*N* = 4, 500^ = 0.15 for the rmGPCM-3 and mPCM-3, respectively), the latent variances (*RMdSE*^*N* = 1, 000^ ≤ 0.05 and *RMdSE*^*N* = 4, 500^ ≤ 0.03 for both models), and the discrimination parameters (*RMdSE*^*N* = 1, 000^ = 0.27 and *RMdSE*^*N* = 4, 500^ = 0.14 for the rmGPCM-3). In this data condition, only delta beta parameters were estimated more precisely than in the reference condition, indicating that sufficient category-frequencies positively affect the accuracy of threshold parameter estimates. Coincidently, in the conditions with a few response categories, a perfectly matched order of delta beta parameters to that of a corresponding population model was found by means of Spearman's rank correlation (for details, see Table S10). In contrast, in the test conditions with many response categories, the result was better for a long test than found in the reference condition. Specifically, with at least *N* = 1,000 cases, a high concordance in the order of the delta beta parameters was reached (with the exception of a class g3, *r*_*s*_ ≥ 0.80 for the rmGPCM-3 and mPCM-3).

**Figure 5:**
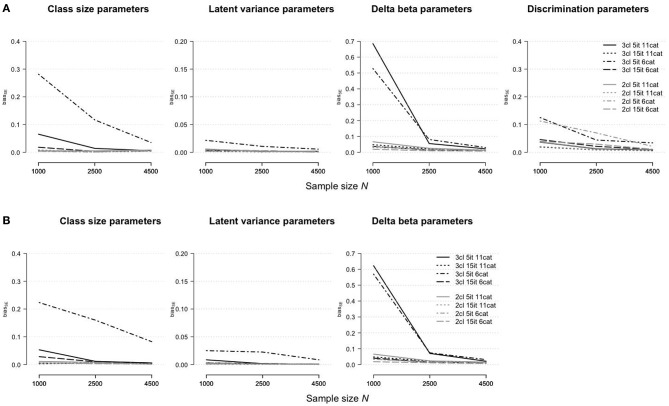
Bias of standard error estimates for parameter estimates in **(A)** the rmGPCM-3 and **(B)** the mPCM-3 under further data conditions.

Figure 5 illustrates the accuracy of the standard error estimates for the rmGPCM-3 (part A) and the mPCM-3 (part B) under different test and sample size conditions. For long tests, the standard errors of all model parameters were accurately estimated ($bias_{se}^{N=1,000}$ ≤ 0.05 and $bias_{se}^{N=4,500}$ ≤ 0.02 for both models). The largest bias was found in the standard errors in the condition of a short test with a few response categories (for class-size parameters, $bias_{se}^{N=1,000}$ = 0.28 and 0.22 and $bias_{se}^{N=4,500}$ = 0.03 and 0.08 for the rmGPCM-3 and mPCM-3, respectively; for delta beta parameters, $bias_{se}^{N=1,000}$ = 0.53 and 0.57 and $bias_{se}^{N=4,500}$ = 0.03 for the rmGPCM-3 and mPCM-3, respectively), suggesting that with regard to model complexity (three-class mixture), individual response vectors did not provide enough information required for reliable unmixing the sample into latent classes and accurate estimation of item parameters (especially with small samples).

**Figure 6:**
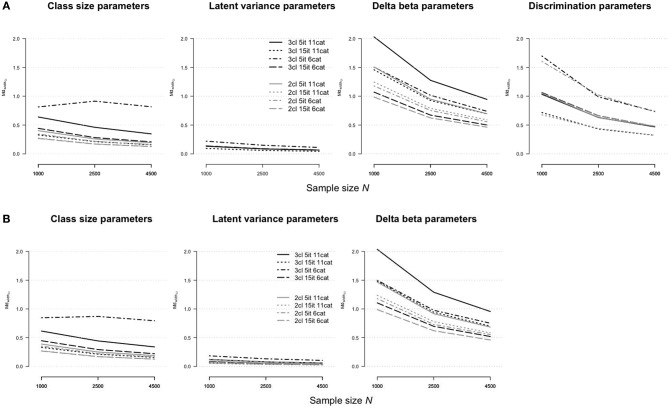
Width of confidence interval for parameter estimates in **(A)** the rmGPCM-3 and **(B)** the mPCM-3 under further data conditions.

Figure 6 shows the widths of confidence intervals for the parameter estimates of the rmGPCM-3 (part A) and the mPCM-3 (part B) under different test and sample size conditions. The width of a confidence interval depends on size of standard errors of a particular parameter type found across replications. We found similar tendencies, as reported above for parameter estimates (using the *RMdSE*), indicating that the standard errors of accurately estimated parameters were small. Compared to the reference condition, the standard errors of all parameter types were smaller for long tests and larger for a short test with few categories (for class-size parameters, $Md_{{width}_{CI}}^{N=1,000}$ = 0.81 and 0.85 for the rmGPCM-3 and the mPCM-3, respectively; for latent variances, $Md_{{width}_{CI}}^{N=1,000}$ = 0.22 and 0.18 for the rmGPCM-3 and the mPCM-3, respectively; for discrimination parameters, $Md_{{width}_{CI}}^{N=1,000}$ = 1.70 and $Md_{{width}_{CI}}^{N=4,500}$ = 0.74 for the rmGPCM-3). In the last condition, the standard errors of delta beta parameters were exceptionally smaller than in the condition of a short test with many categories ($Md_{{width}_{CI}}^{N=1,000}$ = 1.50 and $Md_{{width}_{CI}}^{N=4,500}$ ≤ 0.75 for both models, respectively). Coincidently, the model parameter showed good coverage (≥0.90) even with a small sample under the conditions of long tests (see the upper part of Table 6). Exceptions were found for the class-size parameters of both models at *N* = 1,000 (≥0.88) and the latent variances at *N* = 2,500 (0.87) in the condition of a long test with few categories, suggesting a reduced classification accuracy of complex IRT models when modeling data of a small sample in which responses were assessed with a short rating scale. In the condition of a short test with few categories, sufficient coverage rates were observed for all parameter types of the rmGPCM-3 at *N* = 4,500. In contrast, the class-size parameters of the mPCM3 were not covered sufficiently even with a large sample (0.87). These results were poorer than found for the reference condition. (Details on the distributions of accuracy indices for the rmGPCM-2 and the mPCM-2 are provided in Tables S11–S13).

**Table 6:** Mean coverage for the parameters of the rmGPCM and the mPCM under further data conditions.

	**Condition**	**5 items 11 cat**.			**15 items 11cat**.			**5 items 6 cat**.			**15 items 6 cat**.		
	***N***	**1,000**	**2,500**	**4,500**	**1,000**	**2,500**	**4,500**	**1,000**	**2,500**	**4,500**	**1,000**	**2,500**	**4,500**
**True latent mixture**	**Parameter type**												
Three-class mixture	**rmGPCM-3**												
	π_*g*_				0.95	0.96	0.94	**0.57**	**0.77**	0.90	**0.89**	0.96	0.91
	λ_*g*_				0.95	0.96	0.90	**0.89**	0.94	0.91	0.96	0.98	0.95
	${\Delta \beta }_{0sg}^{i}$ ^a^				0.96	0.95	0.95	**0.81**	**0.87**	0.93	0.95	0.95	0.95
	λ^*i*a^				0.96	0.94	0.93	**0.56**	**0.78**	0.90	0.96	0.98	0.94
	**mPCM-3**												
	π_*g*_				0.95	0.95	0.96	**0.74**	**0.80**	**0.87**	**0.88**	0.94	0.94
	λ_*g*_				0.93	0.95	0.96	0.96	0.90	0.93	0.93	**0.87**	0.95
	${\Delta \beta }_{0sg}^{i}$ ^a^				0.96	0.95	0.95	0.92	0.93	0.95	0.94	0.95	0.95
Two-class mixture	**rmGPCM-2**												
	π_*g*_	0.98	0.90	0.92	0.92	0.94	0.96	0.96	0.92	0.92	0.96	0.92	0.92
	λ_*g*_	0.93	0.94	0.94	0.98	0.94	0.96	0.98	0.90	0.96	0.93	0.90	0.95
	${\Delta \beta }_{0sg}^{i}$ ^a^	0.96	0.96	0.96	0.96	0.96	0.95	0.96	0.96	0.95	0.96	0.96	0.96
	λ^*i*a^	0.96	0.95	0.96	0.96	0.96	0.95	0.97	0.92	0.95	0.95	0.94	0.93
	**mPCM-2**												
	π_*g*_	0.98	0.90	0.98	0.92	0.94	0.96	0.92	0.94	0.90	0.98	0.94	0.94
	λ_*g*_	0.95	0.99	0.94	0.98	0.98	0.96	0.97	0.95	0.96	0.94	0.93	0.92
	${\Delta \beta }_{0sg}^{i}$ ^a^	0.96	0.95	0.96	0.96	0.95	0.95	0.97	0.95	0.95	0.96	0.95	0.95

*π_g_, latent class-size parameter; λ_LGg_, estimate of the variance of the class-specific latent trait variable; ${\Delta \beta }_{0sg}^{i}$, delta beta parameter; λ^i^, item discrimination parameter*.

a *Mean coverage is reported for this parameter type*.

*Coverage rate under 0.90 is shown in bold*.

#### Combined With a True Two-Class Mixture

When the data represented a simple latent mixture, the rmGPCM-2 and mPCM-2 produced no estimation problems (see Table S9). Furthermore, under all data conditions, these models had a general tendency to produce more accurate estimates, as reported above for the complex mixture IRT models (see Figures 4–6; for details, see also Tables S11–S13). In particular, in the challenging data condition (drawn as a solid gray line), the rmGPCM-2 and mPCM-2 indicated a low bias in the class-size parameters (*RMdSE*^*N* = 1, 000^ = 0.06) and the latent variances (*RMdSE*^*N* = 1, 000^ = 0.02) and a slightly higher bias in the item parameters (for delta beta parameters, *RMdSE*^*N* = 1000^ ≤ 0.26 and *RMdSE*^*N* = 4, 500^ ≤ 0.13 for both models; for discrimination parameters, *RMdSE*^*N* = 1, 000^ = 0.17 and *RMdSE*^*N* = 4, 500^ = 0.07 for the rmGPCM-2). In further data conditions, the parameter estimates showed an equal or superior estimation accuracy [with an exception of the discrimination parameters of the rmGPCM-2 in the condition of a short test with few categories (*RMdSE*^*N* = 1, 000^ = 0.32 and *RMdSE*^*N* = 4, 500^ = 0.14)]. In addition, the simple mixture IRT models could well represent the correct order of the thresholds under different data conditions, regardless of sample size [with an exception of the rmGPCM-2 in the challenging data condition at *N* = 1,000 (≥0.87)]. Moreover, the standard errors of all model parameters were estimated with a low bias ($bias_{se}^{N=1,000}$ ≤ 0.11 for both models). The confidence intervals were small for the class-size parameters ($Md_{{width}_{CI}\;}^{N=1,000}\leq$ 0.40 and $Md_{{width}_{CI}}^{N=4,500}=$ 0.19 for both models) and the latent variances ($Md_{{width}_{CI}\;}^{N=1,000}\leq$ 0.19 and $Md_{{width}_{CI}}^{N=4,500}$ = 0.09 for both models), whereas those were larger for the delta beta parameters, especially in the challenging data condition ($Md_{{width}_{CI}\;}^{N=1,000}\leq$ 1.51 and $Md_{{width}_{CI}}^{N=4,500}\leq$ 0.70 for both models), and for the discrimination parameters of the rmGPCM-2, primarily in the condition of a short test with few categories ($Md_{{width}_{CI}\;}^{N=1,000}$ = 1.60 and $Md_{{width}_{CI}}^{N=4,500}$ = 0.73). All types of parameters of the simple mixture IRT models reached a sufficient coverage rate.

### Model Selection

Table 7 reports the proportion of replications in which the true class solution of the two population models consisting of a true three-class mixture was correctly identified as the best-fitting solution by the examined information criteria as well as their under- or overestimation rate across sample size conditions. (Conditions with a proper performance are marked in bold). Under the challenging data condition, for the rmGPCM, the AIC3 was the best criterion for selecting the three-class solution from medium-sized samples (from *N* = 1,500), followed by the SABIC (from *N* = 2,500). In contrast, the BIC and the CAIC constantly underestimated the true number of classes in the conditions with small and medium-sized samples, but they properly worked primarily with large samples (from *N* = 4,500/5,000, respectively). Whereas the AIC showed a consistent tendency to overestimate the true number of classes (with only 79–84% success rate across sample-size conditions). Referring to the mPCM, the results are similar to those of the rmGPCM. Under the long test conditions, all information criteria (with the exception of the AIC) worked more effectively. Specifically, when the models were applied to the data assessed with a long test with many categories, the AIC3 and SABIC showed 100% success rate from a small sample (*N* = 1,000) and the BIC and CAIC from a medium-sized sample (*N* = 2,500). For modeling data assessed with a long test and few categories, the AIC3 and SABIC could perfectly identify the correct class solution from the medium-sized sample and the BIC and CAIC from large sample size (*N* = 4,500). With regard to a short test with few categories, these information criteria worked unsatisfactorily: for the rmGPCM, only the AIC3 was effective for a large sample (98% success rate); for the mPCM, the AIC3, and SABIC were successful from a medium-sized sample, whereas the BIC and CAIC showed a high success rate from a large sample. Regarding the population models with a simple true mixture, all information criteria (with the exception of the AIC) could perfectly identify the correct number of latent classes under all data conditions (see Table 8). However, the AIC tended to overestimate the correct class solution for complex and simple mixed IRT models (indicating the success rate of 0–98%).

**Table 7:** Model selection for the rmGPCM and the mPCM under the condition of a true three-class mixture.

		**AIC**			**BIC**			**CAIC**			**AIC3**			**SABIC**		
**Condition**	***N***	**g2**	**g3^a^**	**g4**	**g2**	**g3^^a^^**	**g4**	**g2**	**g3^^a^^**	**g4**	**g2**	**g3^^a^^**	**g4**	**g2**	**g3^^a^^**	**g4**
**rmGPCM-3**																
5 items 11 cat.	500	17	77	6	2	0	0	0	0	0	98	2	0	99	1	0
	1,000	0	84	16	99	0	0	83	0	0	37	63	0	91	9	0
	1,500	0	81	19	100	0	0	100	0	0	1	**99**	0	56	44	0
	2,000	0	80	20	100	0	0	100	0	0	0	**100**	0	16	84	0
	2,500	0	79	21	100	0	0	100	0	0	0	**100**	0	2	**98**	0
	3,000	0	82	18	92	8	0	100	0	0	0	**100**	0	0	**100**	0
	3,500	0	81	19	69	31	0	97	3	0	0	**100**	0	0	**100**	0
	4,000	0	78	22	21	79	0	66	34	0	0	**100**	0	0	**100**	0
	4,500	0	82	18	5	**95**	0	40	60	0	0	**100**	0	0	**100**	0
	5,000	0	80	20	0	**100**	0	7	93	0	0	**100**	0	0	**100**	0
15 items 11 cat.	1,000	0	92	8	100	0	0	100	0	0	0	**100**	0	0	**100**	0
	2,500	0	36	64	0	**100**	0	0	**100**	0	0	**100**	0	0	**100**	0
	4,500	0	16	84	0	**100**	0	0	**100**	0	0	**100**	0	0	**100**	0
5 items 6 cat.	1,000	32	24	44	100	0	0	100	0	0	100	0	0	100	0	0
	2,500	0	52	48	100	0	0	100	0	0	68	32	0	100	0	0
	4,500	0	40	60	100	0	0	100	0	0	2	**98**	0	90	10	0
15 items 6 cat.	1,000	0	18	82	100	0	0	100	0	0	6	94	0	68	32	0
	2,500	0	10	90	72	28	0	96	4	0	0	**100**	0	0	**100**	0
	4,500	0	2	98	0	**100**	0	0	**100**	0	0	**100**	0	0	**100**	0
**mPCM-3**																
5 items 11 cat.	500	18	78	4	04	0	0	0	0	0	98	2	0	99	1	0
	1,000	0	86	14	100	0	0	87	0	0	33	67	0	87	13	0
	1,500	0	83	17	100	0	0	100	0	0	1	**99**	0	47	53	0
	2,000	0	82	18	100	0	0	100	0	0	0	**100**	0	12	88	0
	2,500	0	81	19	99	1	0	100	0	0	0	**100**	0	1	**99**	0
	3,000	0	80	20	85	15	0	100	0	0	0	**100**	0	0	**100**	0
	3,500	0	80	20	39	61	0	79	21	0	0	**100**	0	0	**100**	0
	4,000	0	79	21	13	87	0	53	47	0	0	**100**	0	0	**100**	0
	4,500	0	78	22	1	**99**	0	11	89	0	0	**100**	0	0	**100**	0
	5,000	0	82	18	0	**100**	0	3	**97**	0	0	**100**	0	0	**100**	0
15 items 11 cat.	1,000	0	**98**	2	96	4	0	100	0	0	0	**100**	0	0	**100**	0
	2,500	0	42	58	0	**100**	0	0	**100**	0	0	**100**	0	0	**100**	0
	4,500	0	14	86	0	**100**	0	0	**100**	0	0	**100**	0	0	**100**	0
5 items 6 cat.	1,000	0	12	88	100	0	0	100	0	90	0	6	94	76	24	0
	2,500	0	0	100	94	6	0	100	0	0	0	**100**	0	0	**100**	0
	4,500	0	0	100	0	**100**	0	0	**100**	0	0	**100**	0	0	**100**	0
15 items 6 cat.	1,000	0	12	88	100	0	0	100	0	0	6	94	0	76	24	0
	2,500	0	0	100	94	6	0	100	0	0	0	**100**	0	0	**100**	0
	4,500	0	0	100	0	**100**	0	0	**100**	0	0	**100**	0	0	**100**	0

*AIC, Akaike's information criterion; BIC, Bayesian information criterion; CAIC, Consistent Akaike's Information Criterion; AIC3, Akaike Information Criterion; SABIC, sample size adjusted BIC*.

*g2, g3, and g4 indicate a two-class to a four-class solution as the best-fitting model, respectively*.

a *The true class solution*.

*A sufficient proportion of replications selected by a certain information criterion is shown in bold*.

**Table 8:** Model selection for the rmGPCM and the mPCM under the condition of a true two-class mixture.

		**AIC**			**BIC**			**CAIC**			**AIC3**			**SABIC**		
**Conditions**	***N***	**g1**	**g2^^a^^**	**g3**	**g1**	**g2^^a^^**	**g3**	**g1**	**g2^^a^^**	**g3**	**g1**	**g2^^a^^**	**g3**	**g1**	**g2^^a^^**	**g3**
**rmGPCM-2**																
5 items 11 cat.	1,000	0	58	42	0	**100**	0	0	**100**	0	0	**100**	0	0	**100**	0
	2,500	0	48	52	0	**100**	0	0	**100**	0	0	**100**	0	0	**100**	0
	4,500	0	52	48	0	**100**	0	0	**100**	0	0	**100**	0	0	**100**	0
5 items 6 cat.	1,000	0	96	4	0	**100**	0	0	**100**	0	0	**100**	0	0	**100**	0
	2,500	0	64	36	0	**100**	0	0	**100**	0	0	**100**	0	0	**100**	0
	4,500	0	34	66	0	**100**	0	0	**100**	0	0	**100**	0	0	**100**	0
15 items 6 cat.	1,000	0	66	34	0	**100**	0	0	**100**	0	0	**100**	0	0	**100**	0
	2,500	0	68	32	0	**100**	0	0	**100**	0	0	**100**	0	0	**100**	0
	4,500	0	66	34	0	**100**	0	0	**100**	0	0	**100**	0	0	**100**	0
15 items 11 cat.	1,000	0	46	54	0	**100**	0	0	**100**	0	0	**100**	0	0	**100**	0
	2,500	0	26	74	0	**100**	0	0	**100**	0	0	**100**	0	0	**100**	0
	4,500	0	4	96	0	**100**	0	0	**100**	0	0	**100**	0	0	**100**	0
**mPCM-2**																
5 items 11 cat.	1,000	0	70	30	0	**100**	0	0	**100**	0	0	**100**	0	0	**100**	0
	2,500	0	62	38	0	**100**	0	0	**100**	0	0	**100**	0	0	**100**	0
	4,500	0	26	74	0	**100**	0	0	**100**	0	0	**100**	0	0	**100**	0
5 items 6 cat.	1,000	0	90	10	0	**100**	0	0	**100**	0	0	**100**	0	0	**100**	0
	2,500	0	42	58	0	**100**	0	0	**100**	0	0	**100**	0	0	**100**	0
	4,500	0	8	92	0	**100**	0	0	**100**	0	0	**100**	0	0	**100**	0
15 items 6 cat.	1,000	0	72	28	0	**100**	0	0	**100**	0	0	**100**	0	0	**100**	0
	2,500	0	58	42	0	**100**	0	0	**100**	0	0	**100**	0	0	**100**	0
	4,500	0	54	46	0	**100**	0	0	**100**	0	0	**100**	0	0	**100**	0
15 items 11 cat.	1,000	0	26	74	0	**100**	0	0	**100**	0	0	**100**	0	0	**100**	0
	2,500	0	0	100	0	**100**	0	0	**100**	0	0	**100**	0	0	**100**	0
	4,500	0	0	100	0	**100**	0	0	**100**	0	0	**100**	0	0	**100**	0

*AIC, Akaike's information criterion; BIC, Bayesian information criterion; CAIC, Consistent Akaike's Information Criterion; AIC3, Akaike Information Criterion; SABIC, sample-size adjusted BIC*.

*g1, g2, and g3 indicate a one-class to a three-class solution as the best-fitting model, respectively*.

a *The true class solution*.

*A sufficient proportion of replications selected by a certain information criterion is shown in bold*.

## Discussion

The results of the present simulation study are useful for researchers interested in applying mixed polytomous IRT models for analyzing rating scales that are widely used in the social and behavioral sciences. When a rating scale consists of many response categories, it is likely to be confronted with the problem of sparse tables when different items are analyzed together. Therefore, the question of what sample size is required for the proper performance of the model is of high importance. Because only very few simulation studies have been conducted to examine mixed polytomous IRT models in general, and no simulation studies were found that considered the performance of these models under the challenging data condition that is typically observed in survey studies (a short scale with a large number of response categories), this application-oriented simulation study focused on the sample size requirements for two models, the rmGPCM and the mPCM, that are useful for exploring category use when applied to such data. Unlike most previous research, we took data-generating model parameters from an empirical model application in order to ensure ecological validity. We additionally examined what sample size is needed for an application of these models under different data conditions such as a long test with many response categories, a long test with a few response categories, and a short test with a few response categories. Moreover, we varied the complexity of the latent mixture.

### Required Sample Size for the Challenging Data Condition

For the challenging data condition, study results indicated the effectivity of the EM algorithm to achieve a convergent solution for the mixed polytomous IRT models independently of model complexity and sample size. For more complex mixed IRT models as the rmGPCM-3 (as well as for overfitting models like the rmGPCM-4 or the mPCM-4), the NR method often produced non-convergent solutions in all sample size conditions. In contrast, for the more parsimonious model (the mPCM-3), this problem occurred to a small extent and disappeared from the medium-sized sample (*N* = 3,000) on. Because of this failure of the NR method to work well in the context of complex models in the presence of sparse data, it could be recommended for researchers whose intention is to apply the rmGPCM or an other complex model to use only the EM algorithm (Vermunt and Magidson, 2013).

For the best-fitting three-class solution of the rmGPCM and the mPCM, the accuracy of parameter and standard error estimates was evaluated. For both models, all parameter types (class-size parameters, class-specific variances of the latent trait variable, class-specific delta beta parameters, and item discrimination parameters only in the rmGPCM) and their corresponding standard errors mostly indicated the same trends. First, the estimation accuracy of parameters and standard errors improved as sample size increased. Specifically, delta beta parameters mainly showed slight improvement and appropriately reproduced true order within items (*r*_*s*_ > 0.90) from the sample size of 1,500 observations (except for the small class g3 concerning the last point). Precise standard errors (*bias_se_* < 0.10) could be obtained only from *N* = 2,000 on (with the same exception of the class g3). Similar results were observed for discrimination parameters and their standard errors. In turn, class-size parameters and class-specific variances of the latent trait variable and corresponding standard errors were estimated pretty accurately even with small sample sizes. To obtain appropriate coverage rates for these parameters at least 2,500 observations were, however, necessary. That may be explained by narrow confidence intervals due to small standard errors for these parameters compared to those of item parameters. Second, we observed that class-specific parameters and their standard errors are more precisely estimated in the largest class (g2) and less accurately in the small class (g3). For example, we found that for estimating delta beta parameters of the small class appropriately and to reproduce their true order of the population model sample sizes of 3,500 and 4,000 observations are necessary for the rmGPCM-3 and mPCM-3, respectively. An effect of the class size on estimation accuracy has been already pointed out in previous research (Preinerstorfer and Formann, 2012; Cho, 2014). Third, class-specific delta beta parameters and standard errors of the categories preferred in latent classes were estimated more accurately. For example, the first delta beta parameter and its standard error, especially in the ORS class (g2), was extremely biased due to very low expected frequencies of the lower categories. We found that by increasing the sample size, the bias could be partly compensated in the semi-ERS class (g3) but hardly in the ORS class (g2). The crucial relevance of sufficient category frequencies to gain satisfactory estimation accuracy and to avoid boundary and extreme values has been emphasized in previous research on traditional polytomous IRT models (DeMars, 2003; He and Wheadon, 2013). Fourth, discrimination parameters and standard errors of highly discriminating items were more strongly biased.

We conclude from our results that an application of both models with an assumed three-class mixture to short-scale data assessed with many response categories can be reasonable with the sample size of at least 2,500 observations. Compared to bias statistics from previous research, the estimation accuracy primarily of delta beta parameters of both models in this simulation study was somewhat lower. However, in contrast to other simulation studies, the present study is based on empirically found parameters as true model parameters, which include unordered thresholds, nearly located parameters on the latent continuum, and some extreme parameters, as it is often the case in the real research studies. Moreover, due to the rating scale with many response categories, both models include many delta beta parameters within an item to be estimated. These specifics make the present simulation study unique and its results relevant for applied research. Nevertheless, researchers should be aware of the problem of low category frequencies that will probably occur in the context of the considered data situations and cause estimation problems (in form of boundary, extreme, and inaccurate parameter estimates) that can hardly be remedied only by increasing the sample size. A widespread way of dealing with this problem is to collapse a category with few responses into one of adjacent categories. However, it may lead to a loss of trait information and reduce the accuracy of latent trait estimates (Wetzel and Carstensen, 2014). Also, we discourage practitioners to use a small sample size of fewer than 1,500 observations under which both mixture polytomous IRT models were especially unsuccessful in providing less biased estimates.

### Required Sample Size for Further Data Conditions

Under further data conditions considered, the two complex mixture IRT models (with a true three-class mixtures) had no estimation problems and indicated similar trends in estimation accuracy with varying sample sizes. With a medium-sized sample (*N* = 2,500), the rmGPCM-3 and the mPCM-3 performed better when applied to long tests than under the challenging data condition. Thus, an enlargement of test length improves the accuracy of estimates. With a small sample (*N* = 1,000), both models mainly indicated satisfactory accuracy of estimates when long tests were used, with the exception of their item parameters which were slightly larger biased. Moreover, for the rmGPCM-3, a reduced number of response categories, even with a long test, could impair the classification accuracy resulted from the model application due to an insufficient coverage rate of class-size parameters with a small sample. This suggests that using a rating scale with few response categories can limit a variety of individual response vectors compared to rating scales with many response categories. In contrast, when applied to a short test with few categories, the rmPCM-3 and the mPCM-3 showed poorer performance. This test condition is more challenging than a short test with many categories and, therefore, required large samples (at least *N* = 4,500). Furthermore, the simple mixture IRT models (with two latent classes) generally performed well under all data conditions examined even with a small sample. However, when applied to long tests, the rmGPCM-2 and mPCM-2 produced a slightly larger bias in the item parameter estimates and their standard errors. Moreover, for the rmGPCM-2 applied to the data which were assessed with a short test and few categories, we would recommend using a medium-sized sample due to biased discrimination parameters found with a small sample. Therefore, this result confirmed that a complex latent mixture is a further crucial factor for estimation accuracy.

### Effectivity of Information Criteria

The last focus of this work was to examine five information criteria concerning their effectiveness to detect the true class solution of the rmGPCM and the mPCM: AIC, BIC, CAIC, AIC3, and SABIC. These information criteria worked differently depending on the latent mixture. When a complex latent mixture was present in the data, for both models, the best result was found for the AIC3, following by the SABIC. The AIC3 showed 99% accuracy of *N* = 1,500 for the challenging data condition and 100% accuracy of *N* = 1,000 for a long test with many response categories and of *N* = 2,500 for a long test with a few response categories. The SABIC indicated 98% accuracy of *N* = 2,500 for the challenging data condition and worked identically well as the AIC3 in the conditions of long tests. This is consistent with the research in the field of finite mixture modeling, reporting that these information criteria are effective for identifying complex latent mixtures (above two classes) with sufficiently small sample size (Fonseca, 2010; Yu and Park, 2014; Choi et al., 2017). But these results are in opposite to the research evidence suggesting the BIC and the CAIC to be as favorites for model selection applied to mixed IRT models (e.g., Li et al., 2009; Cho, 2014). The present simulation study indicated that these information criteria generally underestimated the true number of classes and worked well only for large samples (of *N* = 4,500/5,000, respectively) under the challenging data condition and for a long test with a few response categories. However, the BIC and CAIC were more effective in the conditions of a long test with many response categories (100% success of *N* = 2,500). In general, the results of this study showed that in the context of mixed polytomous IRT models the more effective information criteria are those that do not or slightly penalize the sample size used for the model application. All four information criteria were unable to identify the true latent mixture when data were assessed with a short test and a few response categories (with the exception of the AIC3 of *N* = 4,500), indicating that such data possess insufficient variety that is required to correctly identify the true latent mixture. However, all four information criteria worked perfectly in all data conditions examined when data comprised a simple latent mixture (two-class mixture). Concerning the AIC, this information criterion selected the correct class solution on average only in 54% of all cases and otherwise preferred an overparameterized model solution. This result is consistent with previous research on mixed dichotomous IRT models (e.g., Cho et al., 2013). Based on our results, we primarily recommend to use the AIC3 and the SABIC for selecting the best-fitting solution of the rmGPCM and the mPCM.

## Limitations and Future Research

The generalization of the reported simulation results is limited due to the specificity of the data situation considered and the latent mixtures (three or two unequally-sized classes with certain category use). In addition, we did not include further useful mixed IRT models (e.g., the mixed NRM, the mixed GPCM with a random response style effect or mixed multidimensional IRT models) because of their high complexity. Future research should expand the range of data conditions, latent mixtures, and mixed polytomous IRT models in accordance with further application fields of mixed IRT approach. For example, researchers may examine how specific features of a latent mixture (e.g., class sizes, similarity of class-specific item parameters, and the interaction of these factors) would affect the effectivity of information criterion for selecting the true class solution.

In general, simulation results depend on the used estimation method. In the present simulation study, model parameters were estimated by means of the maximum likelihood estimation method, which is implemented in Latent GOLD. However, more and more recent studies on mixed IRT models use the Bayesian estimation method, which is also flexible for model restrictions and extension. Therefore, future research should focus on the comparison of two estimation methods as previous studies on mixed dichotomous IRT models has indicated benefits of the latter method concerning parameter estimation bias and classification accuracy for short scales, small sample sizes, and complex latent mixtures (e.g., Finch and French, 2012).

Future studies should examine whether including external covariates into mixed polytomous IRT models may improve correct identification of the underlying structure and accuracy of class assignments and parameter estimates, especially when data conditions or latent mixtures are challenging. Empirical evidence on this issue has been shown in the context of mixed dichotomous IRT models (see Smit et al., 2000; De la Torre and Hong, 2010; Dai, 2013) but not yet for mixed polytomous IRT models.

## Conclusion

The current application-oriented simulation study was aimed at identifying the required sample size for the mixed one- and two-parameter IRT models for polytomous data (rmGPCM, mPCM) and investigating diverse information criteria concerning their capacity to correctly detect the best-fitting model solution. Focusing on the specific data situation present in panel surveys by assessing aspects of life satisfaction with short scale and many response categories and on the latent mixture of typical category use patterns in that context, this simulation study produced results suggesting that two models exhibited similar trends of estimation accuracy at manipulated sample sizes. Under the challenging data conditions and a complex latent mixture, the sample size of fewer than 1,500 respondents was insufficiently small, and a sample size of 2,500 respondents seemed to be sufficient. A further increase of the sample size had a positive effect on the estimation accuracy, especially in the small class, but was hardly helpful for extremely biased item parameters and standard errors arising in the case of low-frequency categories. In particular, the mixed two-parameter IRT model (rmGPCM) indicated more estimation problems (in form of non-convergence of the Newton-Raphson algorithm, occurrence of extreme parameter estimates, and boundary standard error estimates) due to insufficient responses of few categories as the mixed one-parametric IRT model did. However, increasing test length can prevent estimation problems and improve estimation accuracy even with a smaller sample. The same is valid when data represent a simple latent mixture. The latent mixture can, however, be determined only by the model application. Of information criteria, the AIC3, followed by the SABIC, performed better compared to the BIC and the CAIC.

## Data Availability Statement

The datasets generated for this study are available on request to the corresponding author.

## Author Contributions

TK and ME developed the concept and design of the simulation study. TK was responsible for carrying out simulations, analyses, and wrote the manuscript. ME and CC revised the content critically.

### Conflict of Interest

The authors declare that the research was conducted in the absence of any commercial or financial relationships that could be construed as a potential conflict of interest.
