# Supplementary material for: Sample Size Requirements for Applying Mixed Polytomous Item Response Models: Results of a Monte Carlo Simulation Study
Source: Front Psychol. 2019 Nov 13;10:2494. doi: 10.3389/fpsyg.2019.02494 (PMC6863808; doi:10.3389/fpsyg.2019.02494)
Supplement: Supplementary file 1 [file Presentation_1.pdf]

Supplementary Material

Sample Size Requirements for Applying Mixed Polytomous Item Response Models: Results of a Monte Carlo Simulation Study

Tanja Kutscher*, Michael Eid, Claudia Crayen

*** Correspondence:** Tanja Kutscher tanja.kutscher@lifbi.de

Table S1. *Convergence rates of the EM algorithm and the Newton-Raphson algorithm, the number of required iterations, boundary values and improper solutions, and the mean classification probability for the rmGPCM under the condition of a true three-class mixture and a 5-item scale with 11 categories*

N	Conv. EM, %	Md_{EM} ($Range_{EM}$)	Conv. NR, %	Md_{NR} ($Range_{NR}$)	BV_{SE}, % (improper)	$M_{P(Y\|G)}$
			rmGPCM-1			
500	100	25 (2 – 148)	100	4 (1 – 16)	0	1.00
1000	100	25 (14 – 61)	100	4 (3 – 16)	0	1.00
1500	100	22 (11 – 76)	100	4 (2 – 12)	0	1.00
2000	100	21 (12 – 57)	100	4 (2 – 11)	0	1.00
2500	100	22 (12 – 59)	100	4 (2 – 10)	0	1.00
3000	100	22 (13 – 51)	100	4 (3 – 12)	0	1.00
3500	100	21 (2 – 50)	100	4 (2 – 10)	0	1.00
4000	100	22 (13 – 53)	100	4 (2 – 9)	0	1.00
4500	100	20 (13 – 57)	100	4 (2 – 13)	0	1.00
5000	100	21 (14 – 77)	100	4 (2 – 11)	0	1.00
			rmGPCM-2			
500	100	133 (47 – 525)	99.6	8 (3 – 600)	0.4 (0)	0.93
1000	100	88 (31 – 501)	99.8	8 (3 – 600)	0.2 (0)	0.92
1500	100	67 (34 – 380)	100	5 (2 – 79)	0	0.91
2000	100	60 (30 – 377)	100	4 (3 – 43)	0	0.91
2500	100	56 (32 – 180)	100	4 (2 – 26)	0	0.91
3000	100	55 (30 – 231)	100	4 (2 – 17)	0	0.91
3500	100	58 (28 – 250)	100	4 (2 – 40)	0	0.91
4000	100	51 (27 – 227)	100	4 (2 – 33)	0	0.91
4500	100	56 (30 – 174)	100	3 (2 – 37)	0	0.91
5000	100	51 (25 – 118)	100	4 (2 – 39)	0	0.91
			rmGPCM-4			
500	100	637 (274 – 5396)	50.2	591 (6 – 600)	49.8 (9)	0.87
1000	100	685 (291 – 2536)	35.0	600 (6 – 600)	64.8 (5)	0.82
1500	100	788 (296 – 2931)	26.0	600 (7 – 600)	73.8 (14)	0.80
2000	100	856 (305 – 3803)	22.6	600 (7 – 600)	77.2 (15)	0.79
2500	100	833 (258 – 3975)	22.8	600 (7 – 600)	77.0 (10)	0.78
3000	100	915 (310 – 3463)	22.8	600 (7 – 600)	77.2 (12)	0.77

3500	100	923 (346 – 3478)	15.8	600 (7 – 600)	83.8 (15)	0.77
4000	100	931 (265 – 3151)	17.0	600 (5 – 600)	83.0 (14)	0.77
4500	100	938 (328 – 5614)	15.0	600 (6 – 600)	85.0 (6)	0.76
5000	100	952 (316 – 3744)	12.6	600 (7 – 600)	87.4 (10)	0.76

Notes. *N*: sample size condition. Conv.EM: convergence rate of the EM algorithm. Md_{EM} ($Range_{EM}$): median (range) of iterations required to reach a convergent solution of the EM algorithm. Conv.NR: convergence rate of the Newton-Rapson algorithm. Md_{NR} ($Range_{NR}$): median (range) of iterations required to reach a convergent solution of the Newton-Rapson algorithm (Note, solutions with 600 iterations did not converge). BV_{SE} (improper): proportion of replications with boundary values (the number of replications with an improper solution). $M_{P(Y|G)}$: mean classification probability.

Table S2. *Convergence rates of the EM algorithm and the Newton-Raphson algorithm, the number of required iterations, boundary values and improper solutions, and the mean classification probability for the mPCM under the condition of a true three-class mixture and a 5-item scale with 11 categories*

N	Conv. EM, %	Md_{EM} ($Range_{EM}$)	Conv. NR, %	Md_{NR} ($Range_{NR}$)	BV_{SE}, % (improper)	$M_{P(Y\|G)}$
			mPCM-1			
500	100	16 (7 – 37)	100	3 (2 – 9)	0	1.00
1000	100	15 (5 – 28)	100	3 (2 – 9)	0	1.00
1500	100	15 (6 – 33)	100	3 (2 – 7)	0	1.00
2000	100	14 (5 – 28)	100	3 (2 – 8)	0	1.00
2500	100	14 (7 – 26)	100	3 (2 – 8)	0	1.00
3000	100	14 (7 – 31)	100	3 (2 – 7)	0	1.00
3500	100	14 (7 – 32)	100	3 (2 – 9)	0	1.00
4000	100	13 (7 – 28)	100	3 (2 – 8)	0	1.00
4500	100	13 (7 – 32)	100	3 (2 – 9)	0	1.00
5000	100	13 (7 – 35)	100	3 (2 – 7)	0	1.00
			mPCM-2			
500	100	129 (44 – 666)	99.2	7 (4 – 600)	0.8 (0)	0.93
1000	100	84 (27 – 609)	99.8	8 (2 – 600)	0.2 (0)	0.92
1500	100	67 (29 – 586)	100	5 (2 – 18)	0	0.92
2000	100	56 (25 – 437)	100	4 (2 – 14)	0	0.92
2500	100	53 (30 – 174)	100	4 (3 – 15)	0	0.92
3000	100	52 (28 – 342)	100	4 (2 – 18)	0	0.91
3500	100	51 (28 – 216)	100	4 (2 – 19)	0	0.92
4000	100	48 (26 – 140)	100	4 (2 – 20)	0	0.91
4500	100	51 (27 – 132)	100	4 (2 – 32)	0	0.91
5000	100	47 (26 – 169)	100	3 (2 – 21)	0	0.91
			mPCM-4			
500	100	493 (196 – 2370)	71.4	10 (6 – 600)	28.4 (1)	0.87
1000	100	609 (234 – 2500)	64.0	12 (6 – 600)	36.0 (2)	0.83
1500	100	609 (185 – 4861)	66.6	10 (5 – 600)	33.0 (0)	0.81
2000	100	716 (244 – 2750)	67.2	10 (6 – 600)	32.8 (0)	0.81
2500	100	729 (277 – 4202)	67.2	9 (6 – 600)	32.8 (4)	0.80
3000	100	763 (216 – 3899)	64.6	10 (5 – 600)	35.2 (5)	0.79
3500	100	858 (287 – 4013)	62.8	13 (4 – 600)	37.2 (3)	0.79
4000	100	889 (253 – 5391)	69.6	10 (3 – 600)	30.2 (2)	0.79
4500	100	928 (259 – 6080)	68.0	10 (4 – 600)	32.0 (0)	0.79
5000	100	980 (269 – 3736)	65.8	10 (4 – 600)	34.0 (3)	0.79

Notes. N: sample size condition. Conv.EM: convergence rate of the EM algorithm. Md_{EM} ($Range_{EM}$): the median (range) of iterations required to reach a convergent solution of the EM algorithm. Conv.NR: convergence rate of the Newton-Rapson algorithm. Md_{NR} ($Range_{NR}$): median (range) of iterations required to reach a convergent solution of the Newton-Rapson algorithm (Note, solutions with 600 iterations did not converge). BV_{SE} (improper): proportion of replications with boundary values (the number of replications with an improper solution). $M_{P(Y|G)}$: mean classification probability.

Table S3. *Root median squared error for parameter estimates (RMdSE) under the condition of a true three-class mixture and a 5-item scale with 11 categories*

	rmGPCM-3						mPCM-3		
N	Item	λ^i	Class	$\Delta\beta_{0sg^1}$	λ_g	π_g	$\Delta\beta_{0sg^1}$	λ_g	π_g
500	2	0.13 [0.07; 0.22]	1	0.64 [0.30; 1.33]	0.03 [0.01; 0.05]		0.68 [0.29; 1.39]	0.03 [0.01; 0.05]	
	3	0.21 [0.09; 0.38]	2	0.45 [0.21; 0.83]	0.04 [0.02; 0.07]	0.17 [0.08; 0.29]	0.36 [0.17; 0.64]	0.04 [0.02; 0.06]	0.16 [0.08; 0.27]
	4	0.52 [0.21; 0.93]	3	0.76 [0.34; 1.69]	0.04 [0.02; 0.07]	0.23 [0.11; 0.36]	0.80 [0.37; 2.10]	0.05 [0.02; 0.08]	0.22 [0.12; 0.36]
	5	0.31 [0.15; 0.60]							
1000	2	0.08 [0.04; 0.14]	1	0.44 [0.21; 0.78]	0.02 [0.01; 0.04]		0.43 [0.20; .75]	0.02 [0.01; 0.04]	
	3	0.15 [0.07; 0.26]	2	0.28 [0.14; 0.51]	0.03 [0.01; 0.05]	0.14 [0.08; 0.23]	0.24 [0.12; .42]	0.03 [0.01; 0.04]	0.12 [0.05; 0.20]
	4	0.36 [0.18; 0.64]	3	0.52 [0.24; 0.95]	0.03 [0.01; 0.05]	0.19 [0.09; 0.32]	0.56 [0.24; 1.08]	0.04 [0.02; 0.06]	0.18 [0.09; 0.30]
	5	0.22 [0.10; 0.38]							
1500	2	0.07 [0.04; 0.12]	1	0.33 [0.15; 0.58]	0.02 [0.01; 0.03]		0.32 [0.16; 0.57]	0.02 [0.01; 0.03]	
	3	0.12 [0.06; 0.19]	2	0.24 [0.11; 0.41]	0.03 [0.01; 0.04]	0.10 [0.05; 0.17]	0.19 [0.10; 0.33]	0.02 [0.01; 0.03]	0.10 [0.04; 0.17]
	4	0.30 [0.13; 0.51]	3	0.37 [0.17; 0.69]	0.02 [0.01; 0.04]	0.14 [0.07; 0.27]	0.42 [0.20; 0.76]	0.03 [0.02; 0.05]	0.14 [0.07; 0.26]
	5	0.17 [0.08; 0.30]							
2000	2	0.06 [0.03; 0.11]	1	0.27 [0.13; 0.48]	0.02 [0.01; 0.03]		0.28 [0.13; 0.50]	0.01 [0.01; 0.03]	
	3	0.11 [0.05; 0.19]	2	0.19 [0.09; 0.35]	0.02 [0.01; 0.03]	0.08 [0.04; 0.14]	0.17 [0.08; 0.29]	0.02 [0.01; 0.03]	0.09 [0.04; 0.14]
	4	0.25 [0.12; 0.41]	3	0.32 [0.15; 0.55]	0.02 [0.01; 0.03]	0.12 [0.06; 0.20]	0.35 [0.16; 0.65]	0.02 [0.01; 0.04]	0.12 [0.06; 0.21]
	5	0.14 [0.06; 0.25]							
2500	2	0.05 [0.02; 0.09]	1	0.25 [0.12; 0.43]	0.01 [0.01; 0.02]		0.24 [0.11; 0.43]	0.01 [0.01; 0.02]	
	3	0.09 [0.04; 0.16]	2	0.18 [0.09; 0.30]	0.02 [0.01; 0.03]	0.08 [0.03; 0.12]	0.15 [0.07; 0.25]	0.02 [0.01; 0.03]	0.06 [0.03; 0.11]
	4	0.23 [0.10; 0.39]	3	0.28 [0.13; 0.48]	0.02 [0.01; 0.03]	0.11 [0.05; 0.18]	0.31 [0.14; 0.54]	0.02 [0.01; 0.03]	0.10 [0.05; 0.18]
	5	0.13 [0.05; 0.21]							

3000	2	0.05 [0.02; 0.09]	1	0.23 [0.11; 0.39]	0.01 [0.01; 0.02]		0.22 [0.10; 0.38]	0.01 [0.01; 0.02]	
	3	0.09 [0.04; 0.14]	2	0.16 [0.07; 0.27]	0.02 [0.01; 0.03]	0.07 [0.03; 0.12]	0.13 [0.06; 0.22]	0.01 [0.01; 0.02]	0.06 [0.03; 0.10]
	4	0.22 [0.10; 0.37]	3	0.24 [0.11; 0.42]	0.02 [0.01; 0.03]	0.10 [0.05; 0.17]	0.28 [0.12; 0.48]	0.02 [0.01; 0.03]	0.09 [0.05; 0.16]
	5	0.12 [0.05; 0.20]							
3500	2	0.05 [0.02; 0.08]	1	0.21 [0.10; 0.36]	0.01 [0.01; 0.02]		0.20 [0.09; 0.35]	0.01 [0.01; 0.02]	
	3	0.08 [0.04; 0.13]	2	0.15 [0.07; 0.25]	0.01 [0.01; 0.02]	0.06 [0.03; 0.10]	0.13 [0.06; 0.22]	0.01 [0.01; 0.02]	0.05 [0.02; 0.10]
	4	0.17 [0.08; 0.30]	3	0.23 [0.11; 0.39]	0.01 [0.01; 0.02]	0.09 [0.04; 0.15]	0.25 [0.12;.0.42]	0.02 [0.01; 0.03]	0.09 [0.04; 0.15]
	5	0.11 [0.05; 0.18]							
4000	2	0.04 [0.02; 0.08]	1	0.19 [0.09; 0.33]	0.01 [0.01; 0.02]		0.20 [0.09; 0.34]	0.01 [0.01; 0.02]	
	3	0.08 [0.04; 0.13]	2	0.14 [0.06; 0.23]	0.01 [0.01; 0.02]	0.06 [0.03; 0.09]	0.12 [0.06; 0.20]	0.01 [0.01; 0.02]	0.05 [0.02; 0.09]
	4	0.18 [0.09; 0.33]	3	0.22 [0.11; 0.37]	0.01 [0.01; 0.02]	0.09 [0.04; 0.14]	0.23 [0.11; 0.39]	0.02 [0.01; 0.03]	0.09 [0.05; 0.15]
	5	0.11 [0.05; 0.18]							
4500	2	0.04 [0.02; 0.07]	1	0.18 [0.08; 0.31]	0.01 [0; 0.02]		0.19 [0.09; 0.32]	0.01 [0; 0.02]	
	3	0.07 [0.04; 0.12]	2	0.13 [0.06; 0.22]	0.01 [0.01; 0.02]	0.05 [0.03; 0.09]	0.11 [0.05; 0.18]	0.01 [0.01; 0.02]	0.05 [0.03; 0.08]
	4	0.15 [0.06; 0.26]	3	0.19 [0.09; 0.34]	0.01 [0.01; 0.02]	0.08 [0.04; 0.13]	0.21 [0.10; 0.37]	0.01 [0.01; 0.02]	0.08 [0.04; 0.13]
	5	0.10 [0.05; 0.17]							
5000	2	0.04 [0.02; 0.07]	1	0.17 [0.08; 0.30]	0.01 [0.01; 0.02]		0.17 [0.08; 0.30]	0.01 [0.01; 0.02]	
	3	0.07 [0.03; 0.12]	2	0.12 [0.06; 0.21]	0.01 [0.01; 0.02]	0.05 [0.03; 0.08]	0.10 [0.05; 0.18]	0.01 [0.01; 0.02]	0.05 [0.02; 0.08]
	4	0.16 [0.07; 0.29]	3	0.18 [0.09; 0.32]	0.01 [0.01; 0.02]	0.06 [0.03; 0.12]	0.21 [0.10; 0.36]	0.02 [0.01; 0.02]	0.08 [0.03; 0.14]
	5	0.10 [0.04; 0.18]							

Note. [1] Median *RMdSE* for class-specific delta beta parameter estimates.

The first and the third quartiles are reported in square brackets.

Table S4. *Empirical standard deviation of parameter estimates ($SD_{\hat{p}}$) under the condition of a true three-class mixture and a 5-item scale with 11 categories*

	rmGPCM-3						mPCM-3		
N	Item	λ^i	Class	$\Delta\beta_{0sg}$[1]	λ_g	π_g	$\Delta\beta_{0sg}$[1]	λ_g	π_g
500	2	0.22	1	2.35	0.05		2.53	0.05	
	3	0.35	2	1.66	0.07	0.26	1.28	0.06	0.24
	4	1.02	3	2.86	0.06	0.32	3.03	0.08	0.31
	5	0.56							
1000	2	0.14	1	1.38	0.03		1.26	0.03	
	3	0.22	2	0.64	0.04	0.19	0.56	0.04	0.17
	4	0.63	3	1.87	0.04	0.27	2.02	0.06	0.27
	5	0.34							
1500	2	0.11	1	0.82	0.03		0.77	0.03	
	3	0.19	2	0.41	0.04	0.15	0.31	0.03	0.14
	4	0.44	3	1.09	0.03	0.23	1.34	0.04	0.22
	5	0.27							
2000	2	0.10	1	0.46	0.02		0.49	0.02	
	3	0.16	2	0.30	0.03	0.12	0.26	0.02	0.12
	4	0.36	3	0.56	0.03	0.18	0.90	0.04	0.19
	5	0.21							
2500	2	0.08	1	0.41	0.02		0.41	0.02	
	3	0.14	2	0.26	0.03	0.11	0.22	0.02	0.10
	4	0.33	3	0.47	0.02	0.15	0.65	0.03	0.16
	5	0.19							
3000	2	0.08	1	0.35	0.02		0.35	0.02	
	3	0.13	2	0.24	0.02	0.10	0.20	0.02	0.09
	4	0.31	3	0.42	0.02	0.14	0.53	0.03	0.14
	5	0.18							
3500	2	0.07	1	0.32	0.02		0.32	0.02	
	3	0.11	2	0.22	0.02	0.09	0.19	0.02	0.09
	4	0.26	3	0.38	0.02	0.13	0.39	0.02	0.13
	5	0.16							
4000	2	0.07	1	0.30	0.02		0.31	0.02	
	3	0.11	2	0.21	0.02	0.08	0.17	0.02	0.08
	4	0.27	3	0.33	0.02	0.12	0.37	0.02	0.13
	5	0.16							
4500	2	0.06	1	0.28	0.02		0.28	0.01	
	3	0.11	2	0.19	0.02	0.08	0.16	0.02	0.07
	4	0.24	3	0.31	0.02	0.11	0.34	0.02	0.11
	5	0.15							
5000	2	0.06	1	0.26	0.01		0.27	0.01	
	3	0.10	2	0.18	0.02	0.07	0.15	0.02	0.07
	4	0.24	3	0.28	0.02	0.10	0.32	0.02	0.11
	5	0.15							

Note. [1] Median standard deviation of class-specific delta beta parameter estimates.

Table S5. *Bias of standard error estimates (bias$_{se}$) under the condition of a true three-class mixture and a 5-item scale with 11 categories*

	rmGPCM-3						mPCM-3		
N	Item	λ^i	Class	$\Delta\beta_{0sg^1}$	λ_g	π_g	$\Delta\beta_{0sg^1}$	λ_g	π_g
500	2	0.05 [0.02; 0.07]	1	1.73 [0.87; 3.73]	0.01 [0; 0.01]		1.86 [0.79; 3.35]	0.01 [0.01; 0.01]	
	3	0.07 [0.03; 0.11]	2	1.07 [0.30; 3.10]	0.02 [0.01; 0.02]	0.09 [0.07; 0.10]	0.81 [0.11; 2.51]	0.02 [0.02; 0.03]	0.08 [0.06; 0.09]
	4	0.33 [0.20; 0.47]	3	2.26 [1.08; 3.75]	0.01 [0.01; 0.02]	0.12 [0.10; 0.14]	2.50 [1.00; 9.93]	0.03 [0.02; 0.04]	0.11 [0.09; 0.12]
	5	0.15 [0.08; 0.22]							
1000	2	0.02 [0.01; 0.03]	1	0.69 [0.20; 1.57]	0 [0; 0.01]		0.60 [0.14; 1.61]	0 [0; 0.01]	
	3	0.03 [0.01; 0.05]	2	0.24 [0.05; 1.33]	0.01 [0; 0.01]	0.05 [0.04; 0.06]	0.23 [0.04; 1.39]	0.01 [0.01; 0.01]	0.04 [0.03; 0.05]
	4	0.14 [0.08; 0.21]	3	1.21 [0.43; 2.24]	0.01 [0; 0.01]	0.09 [0.07; 0.11]	1.37 [0.39; 2.66]	0.02 [0.01; 0.02]	0.08 [0.07; 0.10]
	5	0.05 [0.03; 0.09]							
1500	2	0.01 [0.01; 0.02]	1	0.26 [0.06; 0.68]	0 [0; 0]		0.26 [0.06; 0.81]	0 [0; 0]	
	3	0.02 [0.01; 0.03]	2	0.09 [0.02; 0.71]	0.01 [0; 0.01]	0.02 [0.02; 0.03]	0.06 [0.02; 0.76]	0.01 [0; 0.01]	0.03 [0.02; 0.04]
	4	0.06 [0.03; 0.01]	3	0.56 [0.14; 1.26]	0 [0; 0.01]	0.06 [0.05; 0.07]	0.68 [0.14; 1.92]	0.01 [0.01; 0.01]	0.05 [0.04; 0.07]
	5	0.04 [0.02; 0.06]							
2000	2	0.01 [0; 0.01]	1	0.08 [0.03; 0.37]	0 [0; 0]		0.10 [0.03; 0.35]	0 [0; 0]	
	3	0.02 [0.01; 0.03]	2	0.04 [0.01; 0.40]	0 [0; 0]	0.02 [0.01; 0.02]	0.03 [0.01; 0.35]	0 [0; 0]	0.02 [0.01; 0.03]
	4	0.04 [0.02; 0.07]	3	0.17 [0.05; 0.74]	0 [0; 0.01]	0.03 [0.02; 0.04]	0.37 [0.07; 1.08]	0.01 [0.01; 0.01]	0.04 [0.02; 0.05]
	5	0.02 [0.01; 0.04]							
2500	2	0.01 [0; 0.01]	1	0.05 [0.02; 0.17]	0 [0; 0]		0.07 [0.03; 0.24]	0 [0; 0]	
	3	0.01 [0.01; 0.02]	2	0.03 [0.01; 0.28]	0 [0; 0]	0.01 [0.01; 0.02]	0.02 [0.01; 0.32]	0 [0; 0]	0.01 [0; 0.01]
	4	0.04 [0.02; 0.06]	3	0.11 [0.03; 0.45]	0 [0; 0]	0.02 [0.01; 0.03]	0.20 [0.04; 0.74]	0 [0; 0.01]	0.03 [0.02; 0.03]
	5	0.02 [0.01; 0.03]							

3000	2	0.01 [0; 0.01]	1	0.04 [0.02; 0.12]	0 [0; 0]		0.04 [0.02; 0.12]	0 [0; 0]	
	3	0.01 [0.01; 0.02]	2	0.02 [0.01; 0.18]	0 [0; 0]	0.01 [0.01; 0.01]	0.02 [0.01; 0.13]	0 [0; 0]	0.08 [0; 0.01]
	4	0.03 [0.02; 0.05]	3	0.07 [0.02; 0.29]	0 [0; 0]	0.02 [0.01; 0.03]	0.11 [0.03; 0.50]	0 [0; 0]	0.02 [0.01; 0.02]
	5	0.02 [0.01; 0.02]							
3500	2	0.00 [0; 0.01]	1	0.03 [0.01; 0.07]	0 [0; 0]		0.03 [0.01; 0.09]	0 [0; 0]	
	3	0.01 [0; 0.01]	2	0.02 [0.01; 0.12]	0 [0; 0]	0.01 [0; 0.01]	0.02 [0.01; 0.14]	0 [0; 0]	0.01 [0; 0.01]
	4	0.02 [0.01; 0.04]	3	0.06 [0.02; 0.19]	0 [0; 0]	0.01 [0.01; 0.02]	0.06 [0.20; 0.27]	0 [0; 0]	0.01 [0.01; 0.02]
	5	0.01 [0.01; 0.20]							
4000	2	0.01 [0; 0.01]	1	0.02 [0.01; 0.05]	0 [0; 0]		0.03 [0.01; 0.06]	0 [0; 0]	
	3	0.01 [0; 0.01]	2	0.02 [0.01; 0.10]	0 [0; 0]	0.01 [0; 0.01]	0.01 [0.00; 0.08]	0 [0; 0]	0 [0; 0.01]
	4	0.03 [0.01; 0.04]	3	0.04 [0.01; 0.11]	0 [0; 0]	0.01 [0.01; 0.01]	0.05 [0.02; 0.25]	0 [0; 0]	0.02 [0.01; 0.02]
	5	0.01 [0.01; 0.02]							
4500	2	0.00 [0; 0.01]	1	0.02 [0.01; 0.05]	0 [0; 0]		0.02 [0.01; 0.05]	0 [0; 0]	
	3	0.01 [0; 0.02]	2	0.02 [0.01; 0.08]	0 [0; 0]	0 [0; 0.01]	0.01 [0.00; 0.07]	0 [0; 0]	0 [0; 0.01]
	4	0.02 [0.01; 0.03]	3	0.03 [0.01; 0.09]	0 [0; 0]	0.01 [0.01; 0.02]	0.04 [0.01; 0.16]	0 [0; 0]	0.01 [0.01; 0.02]
	5	0.01 [0.01; 0.02]							
5000	2	0.00 [0; 0.01]	1	0.02 [0.01; 0.04]	0 [0; 0]		0.02 [0.01; 0.04]	0 [0; 0]	
	3	0.01 [0; 0.01]	2	0.01 [0; 0.06]	0 [0; 0]	0 [0; 0.01]	0.01 [0.00; 0.05]	0 [0; 0]	0.01 [0; 0.01]
	4	0.02 [0.01; 0.04]	3	0.03 [0.01; 0.07]	0 [0; 0]	0.01 [0; 0.01]	0.04 [0.01; 0.11]	0 [0; 0]	0.01 [0.01; 0.02]
	5	0.01 [0.01; 0.02]							

Note. [1] Median $bias_{SE}$ for class-specific delta beta parameters.

The first and the third quartiles are reported in square brackets.

Table S6. *Width of confidence interval for parameter estimates (width$_{CI}$) under the condition of a true three-class mixture and a 5-item scale with 11 categories*

	rmGPCM-3						mPCM-3		
N	Item	λ^i	Class	$\Delta\beta_{0sg^1}$	λ_g	π_g	$\Delta\beta_{0sg^1}$	λ_g	π_g
500	2	0.71 [0.62; 0.88]	1	3.06 [2.17; 4.92]	0.17 [0.15; 0.19]		3.15 [2.22; 5.22]	0.15 [0.14; 0.16]	
	3	1.25 [1.07; 1.50]	2	2.05 [1.35; 4.64]	0.20 [0.17; 0.23]	0.66 [0.62; 0.73]	1.82 [1.24; 3.73]	0.17 [0.15; 0.18]	0.64 [0.60; 0.70]
	4	2.97 [2.34; 3.86]	3	3.15 [2.07; 6.88]	0.19 [0.16; 0.22]	0.80 [0.73; 0.86]	3.40 [2.09; 51.53]	0.20 [0.17; 0.23]	0.80 [0.73; 0.86]
	5	1.79 [1.48; 2.35]							
1000	2	0.51 [0.46; 0.57]	1	2.16 [1.58; 3.07]	0.12 [0.11; 0.13]		2.20 [1.61; 3.10]	0.11 [0.10; 0.11]	
	3	0.88 [0.78; 0.99]	2	1.45 [0.97; 2.84]	0.15 [0.13; 0.16]	0.56 [0.51; 0.61]	1.31 [0.90; 2.53]	0.12 [0.11; 0.13]	0.53 [0.49; 0.56]
	4	2.03 [1.72; 2.48]	3	2.36 [1.60; 3.68]	0.13 [0.12; 0.15]	0.72 [0.66; 0.79]	2.55 [1.62; 4.41]	0.15 [0.13; 0.17]	0.71 [0.65; 0.79]
	5	1.25 [1.09; 1.46]							
1500	2	0.41 [0.38; 0.46]	1	1.77 [1.28; 2.41]	0.10 [0.09; 0.10]		1.78 [1.30; 2.42]	0.09 [0.08; 0.09]	
	3	0.70 [0.64; 0.77]	2	1.21 [0.81; 2.35]	0.12 [0.11; 0.13]	0.48 [0.45; 0.52]	1.07 [0.74; 2.06]	0.10 [0.09; 0.11]	0.45 [0.42; 0.48]
	4	1.65 [1.45; 1.92]	3	1.90 [1.31; 2.76]	0.11 [0.10; 0.11]	0.64 [0.59; 0.70]	2.12 [1.34; 3.36]	0.13 [0.12; 0.14]	0.64 [0.59; 0.70]
	5	1.00 [0.98; 1.12]							
2000	2	0.36 [0.33; 0.39]	1	1.51 [1.11; 2.01]	0.08 [0.08; 0.09]		1.54 [1.13; 2.06]	0.08 [0.07; 0.08]	
	3	0.60 [0.55; 0.66]	2	1.06 [0.71; 2.01]	0.11 [0.10; 0.12]	0.43 [0.40; 0.43]	0.94 [0.64; 1.76]	0.09 [0.08; 0.09]	0.40 [0.38; 0.44]
	4	1.43 [1.26; 1.60]	3	1.67 [1.13; 2.33]	0.10 [0.09; 0.11]	0.58 [0.54; 0.62]	1.84 [1.16; 2.75]	0.11 [0.10; 0.12]	0.59 [0.55; 0.64]
	5	0.85 [0.78; 0.95]							
2500	2	0.32 [0.30; 0.34]	1	1.34 [0.99; 1.77]	0.08 [0.07; 0.08]		1.37 [1.00; 1.80]	0.07 [0.07; 0.07]	
	3	0.54 [0.50; 0.58]	2	0.94 [0.63; 1.80]	0.10 [0.09; 0.10]	0.39 [0.37; 0.41]	0.83 [0.58; 1.58]	0.08 [0.08; 0.08]	0.36 [0.34; 0.38]
	4	1.26 [1.13; 1.41]	3	1.49 [1.02; 2.05]	0.09 [0.08; 0.09]	0.53 [0.50; 0.57]	1.66 [1.04; 2.41]	0.10 [0.09; 0.11]	0.54 [0.50; 0.58]
	5	0.76 [0.70; 0.83]							

3000	2	0.29 [0.27; 0.31]	1	1.23 [0.90; 1.60]	0.07 [0.07; 0.07]		1.25 [0.91; 1.63]	0.06 [0.06; 0.07]	
	3	0.49 [0.46; 0.53]	2	0.86 [0.58; 1.64]	0.09 [0.08; 0.09]	0.36 [0.34; 0.38]	0.76 [0.53; 1.45]	0.07 [0.07; 0.08]	0.34 [0.32; 0.35]
	4	1.15 [1.04; 1.29]	3	1.36 [0.92; 1.84]	0.08 [0.08; 0.09]	0.48 [0.46; 0.51]	1.50 [0.94; 2.16]	0.09 [0.09; 0.10]	0.50 [0.47; 0.53]
	5	0.69 [0.65; 0.75]							
3500	2	0.27 [0.25; 0.28]	1	1.14 [0.84; 1.50]	0.07 [0.06; 0.07]		1.16 [0.85; 1.51]	0.06 [0.06; 0.06]	
	3	0.44 [0.42; 0.48]	2	0.80 [0.54; 1.50]	0.08 [0.08; 0.09]	0.34 [0.32; 0.36]	0.71 [0.49; 1.35]	0.07 [0.07; 0.07]	0.31 [0.30; 0.33]
	4	1.02 [0.94; 1.11]	3	1.25 [0.85; 1.67]	0.08 [0.07; 0.08]	0.45 [0.43; 0.48]	1.36 [0.86; 1.91]	0.08 [0.08; 0.09]	0.45 [0.43; 0.49]
	5	0.64 [0.59; 0.69]							
4000	2	0.25 [0.24; 0.27]	1	1.06 [0.78; 1.36]	0.06 [0.06; 0.06]		1.08 [0.79; 1.39]	0.06 [0.05; 0.06]	
	3	0.43 [0.40; 0.45]	2	0.75 [0.50; 1.42]	0.08 [0.07; 0.08]	0.31 [0.30; 0.32]	0.66 [0.46; 1.25]	0.06 [0.06; 0.07]	0.29 [0.28; 0.31]
	4	1.00 [0.91; 1.08]	3	1.17 [0.80; 1.56]	0.07 [0.07; 0.07]	0.42 [0.40; 0.44]	1.29 [0.80; 1.82]	0.08 [0.08; 0.09]	0.43 [0.41; 0.46]
	5	0.60 [0.56; 0.64]							
4500	2	0.24 [0.23; 0.25]	1	1.01 [0.74; 1.31]	0.06 [0.06; 0.06]		1.02 [0.75; 1.32]	0.05 [0.05; 0.05]	
	3	0.40 [0.37; 0.42]	2	0.71 [0.48; 1.34]	0.07 [0.07; 0.08]	0.30 [0.29; 0.31]	0.62 [0.43; 1.18]	0.06 [0.06; 0.06]	0.27 [0.26; 0.29]
	4	0.91 [0.84; 0.98]	3	1.09 [0.74; 1.45]	0.07 [0.06; 0.07]	0.40 [0.38; 0.42]	1.19 [0.75; 1.66]	0.07 [0.07; 0.08]	0.40 [0.38; 0.42]
	5	0.56 [0.52; 0.61]							
5000	2	0.23 [0.21; 0.24]	1	0.95 [0.70; 1.22]	0.05 [0.05; 0.06]		0.97 [0.71; 1.23]	0.05 [0.05; 0.05]	
	3	0.38 [0.36; 0.40]	2	0.68 [0.45; 1.27]	0.07 [0.07; 0.07]	0.28 [0.27; 0.30]	0.59 [0.41; 1.12]	0.06 [0.05; 0.06]	0.26 [0.25; 0.27]
	4	0.88 [0.82; 0.96]	3	1.04 [0.70; 1.36]	0.06 [0.06; 0.06]	0.38 [0.36; 0.39]	1.15 [0.71; 1.61]	0.07 [0.07; 0.08]	0.39 [0.37; 0.41]
	5	0.54 [0.51; 0.68]							

Note. [1] Median $width_{CI}$ for class-specific delta beta parameters.

The first and the third quartiles are reported in square brackets.

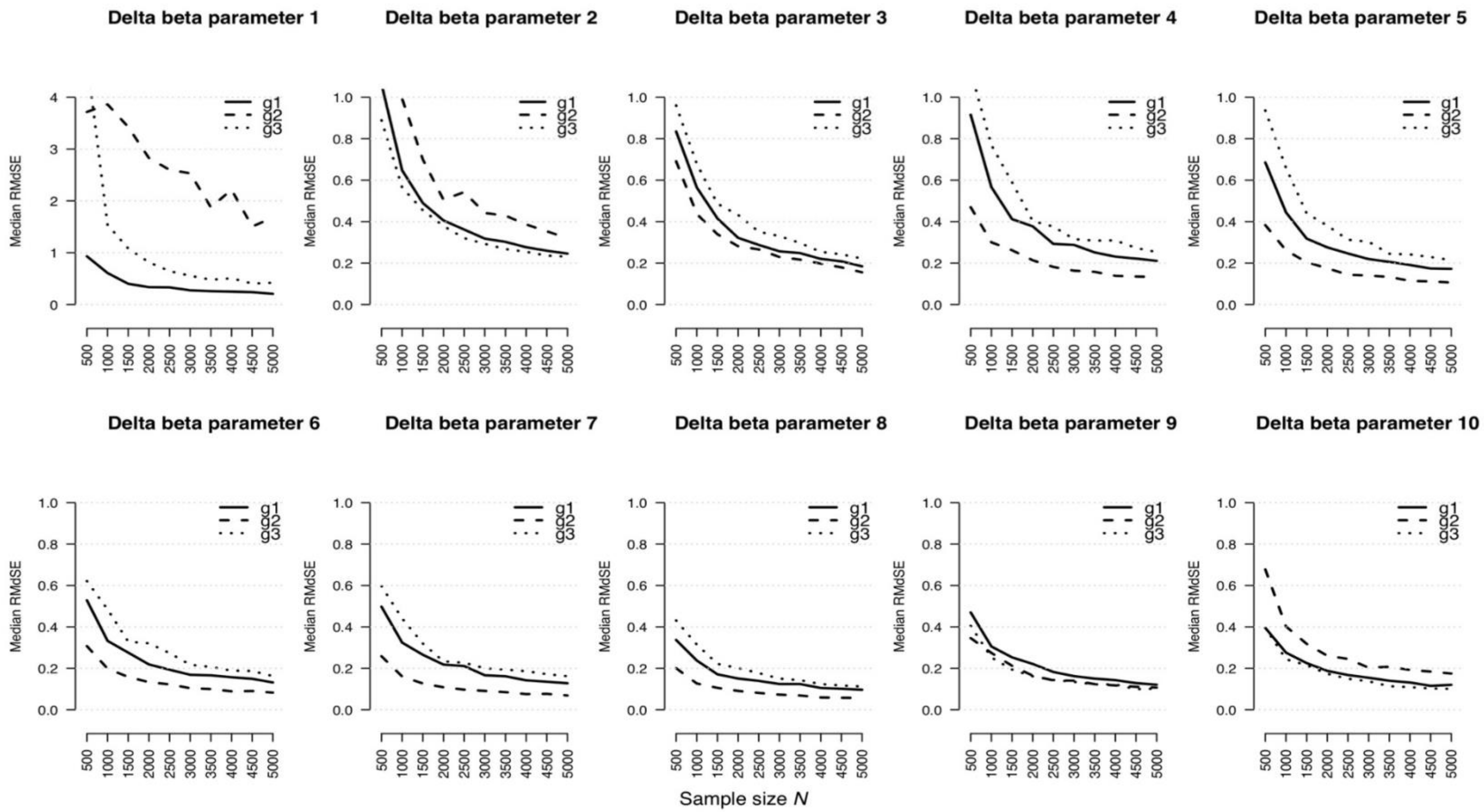

Figure S1. Root median squared error for class-specific delta beta parameter estimates in the rmGPCM-3 *under the condition of a true three-class mixture and a 5-item scale with 11 categories*

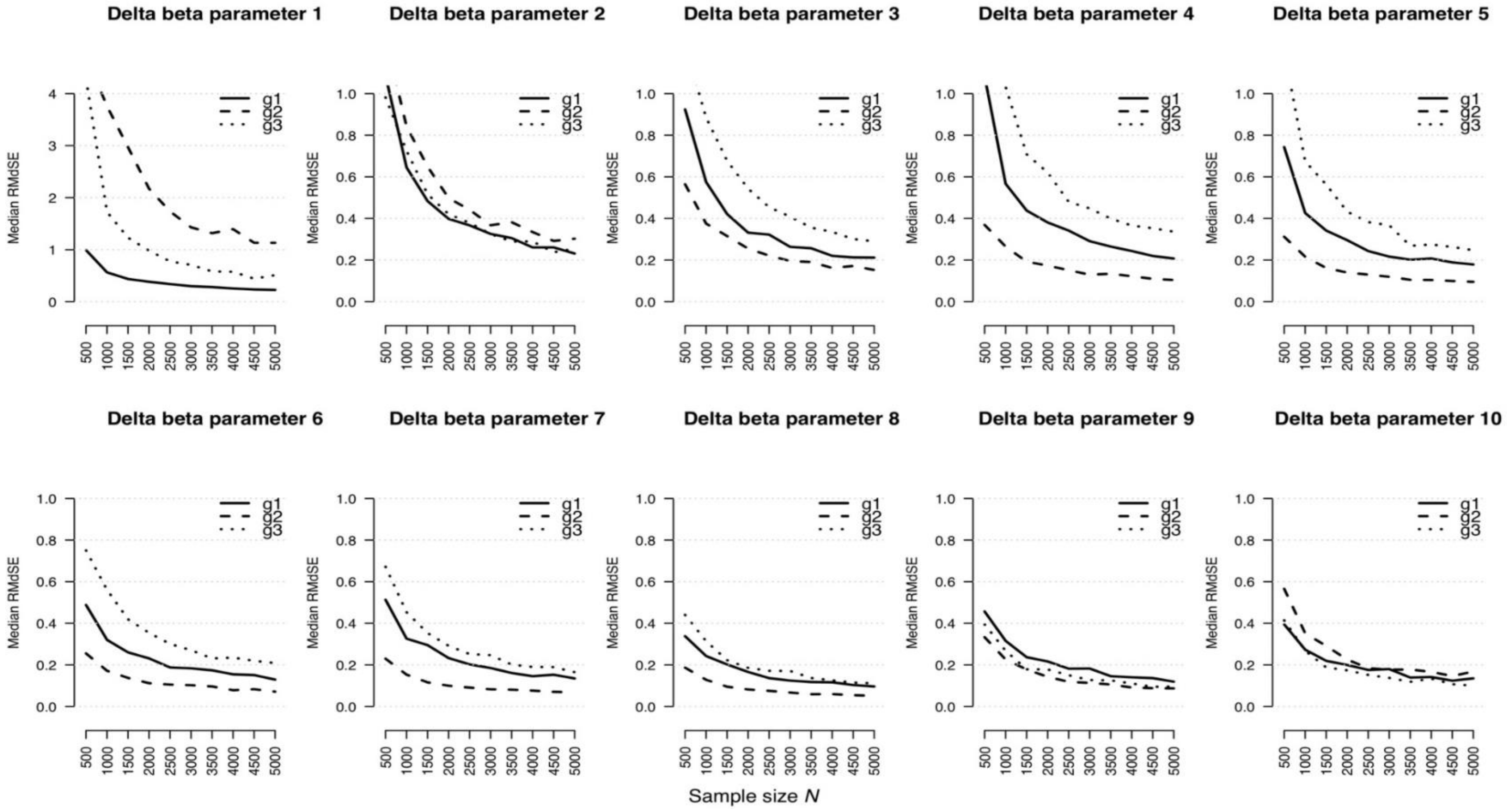

Figure S2. Root median squared error for class-specific delta beta parameter estimates in the mPCM-3 *under the condition of a true three-class mixture and a 5-item scale with 11 categories*

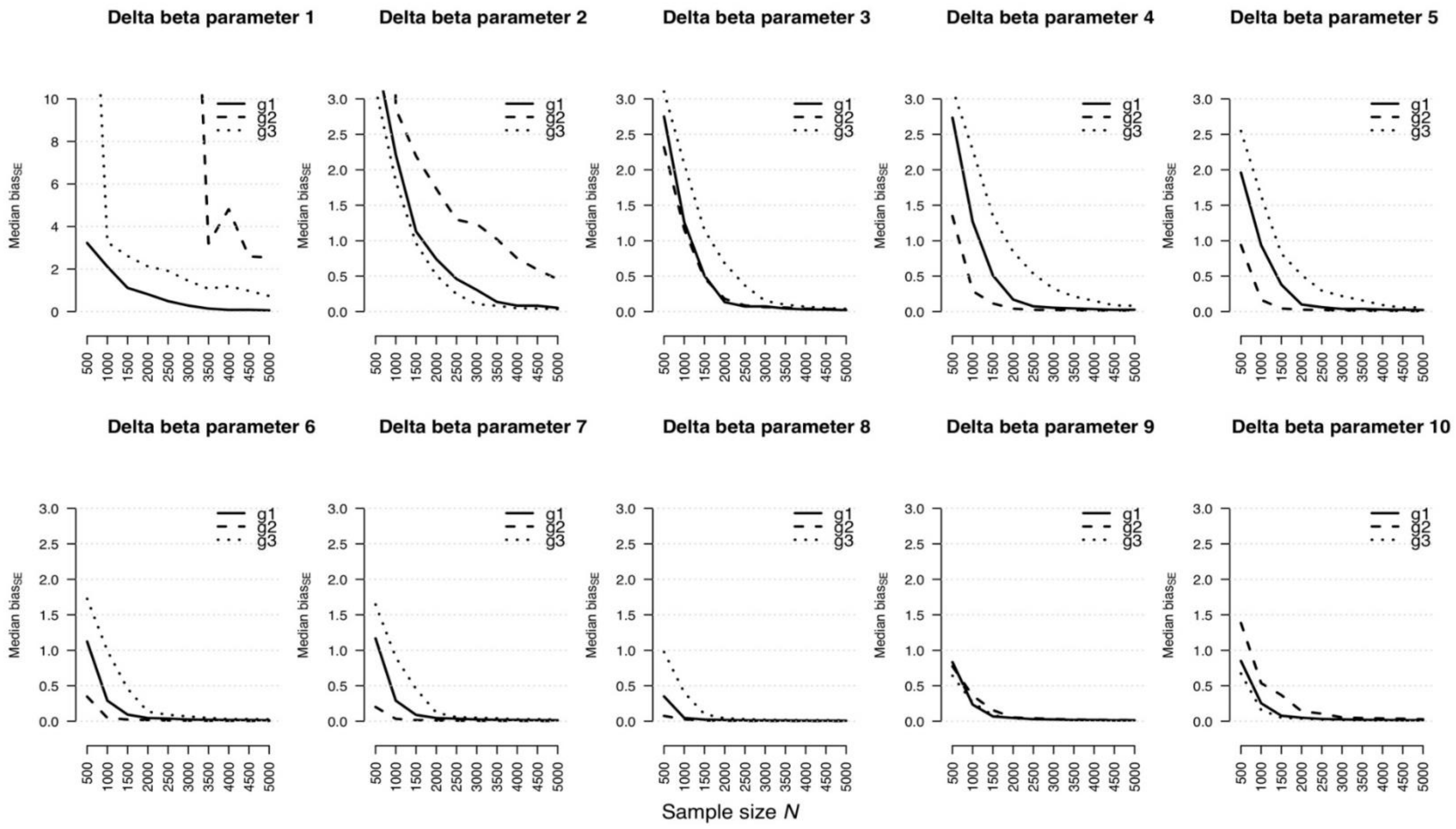

Figure S3. Bias of standard error estimates for class-specific delta beta parameter estimates in the rmGPCM-3 *under the condition of a true three-class mixture and a 5-item scale with 11 categories*

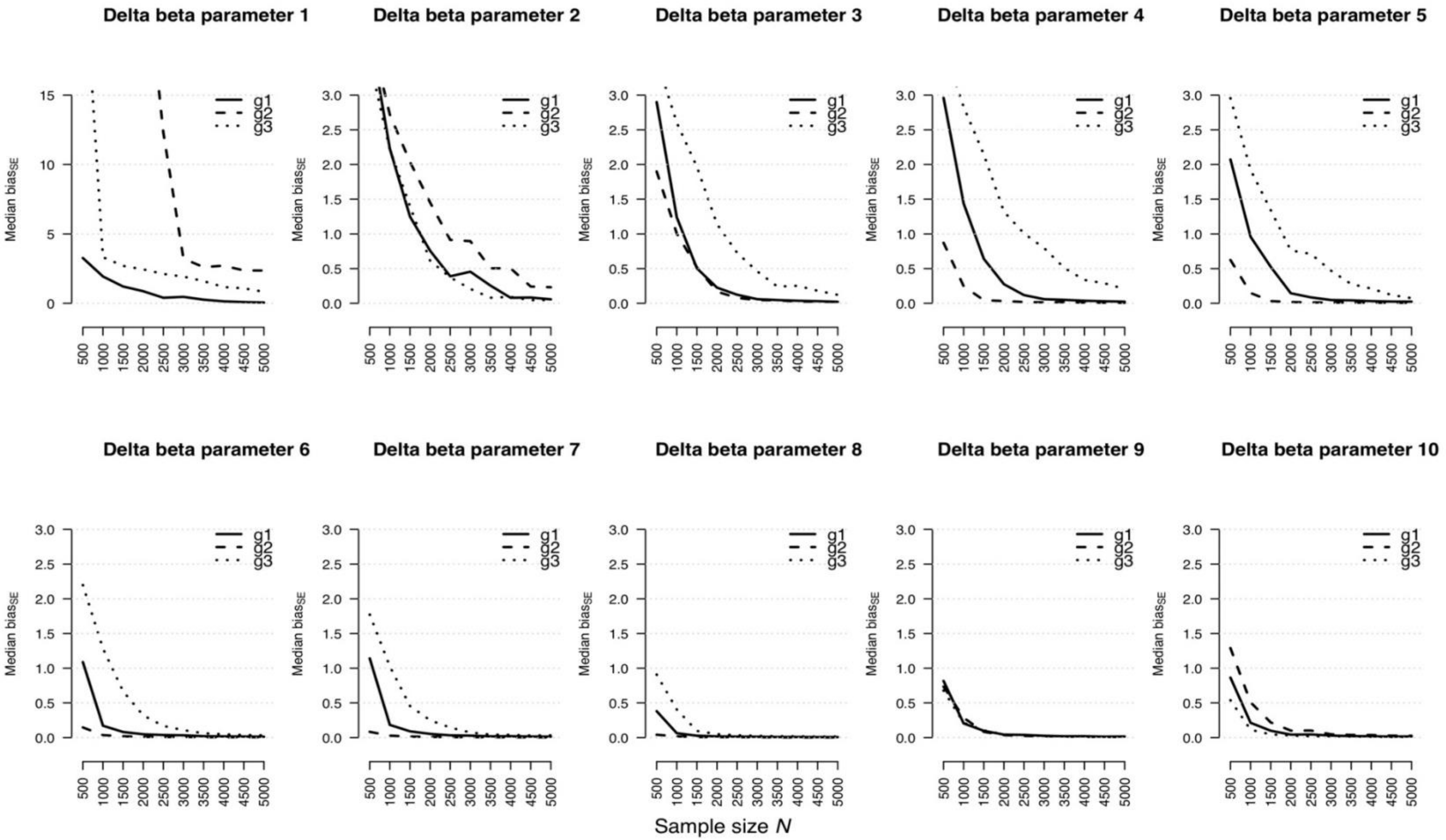

Figure S4. Bias of standard error estimates for class-specific delta beta parameter estimates in the mPCM-3 *under the condition of a true three-class mixture and a 5-item scale with 11 categories*

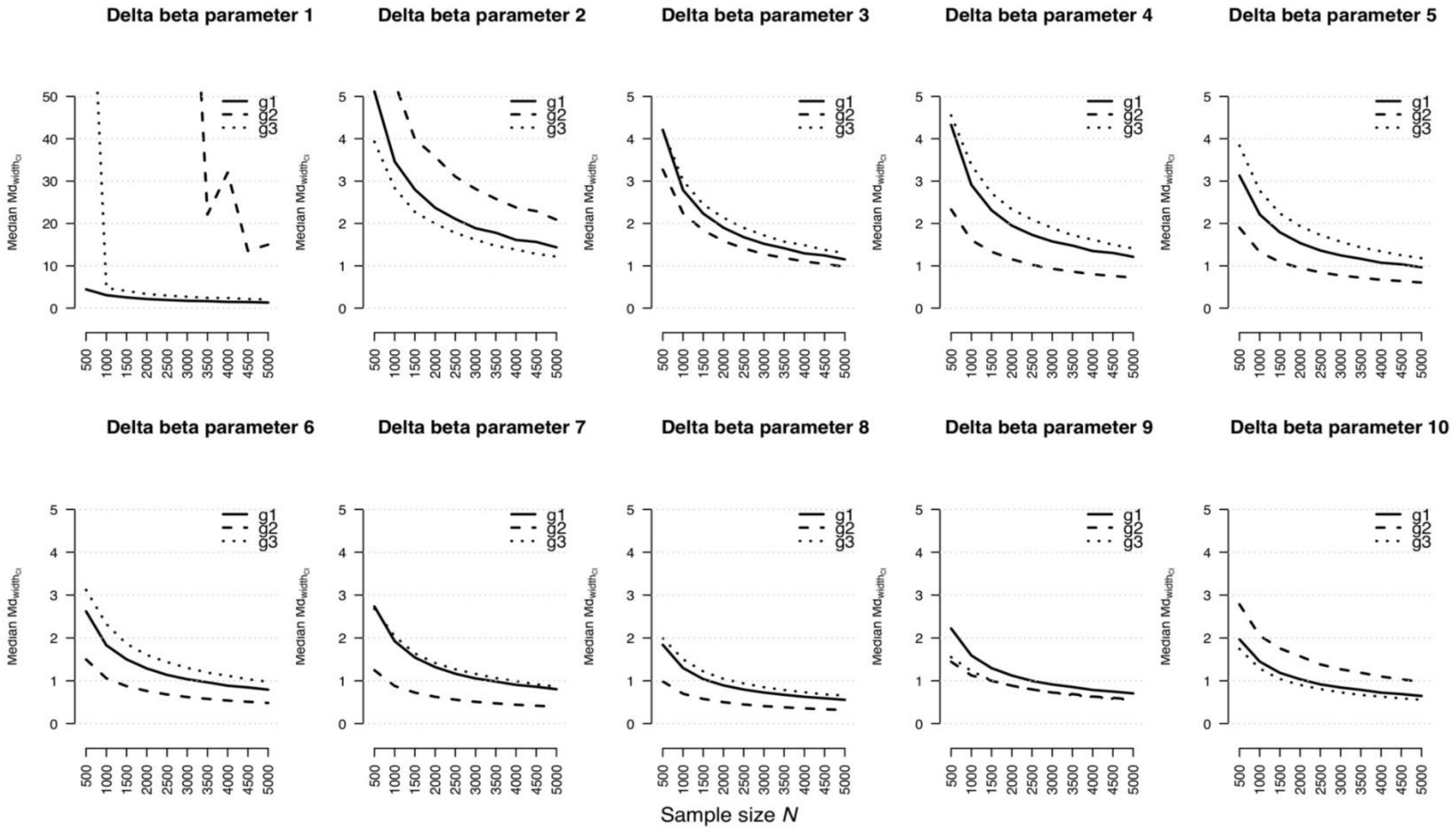

Figure S5. Width of confidence interval for class-specific delta beta parameter estimates in the rmGPCM-3 *under the condition of a true three-class mixture and a 5-item scale with 11 categories*

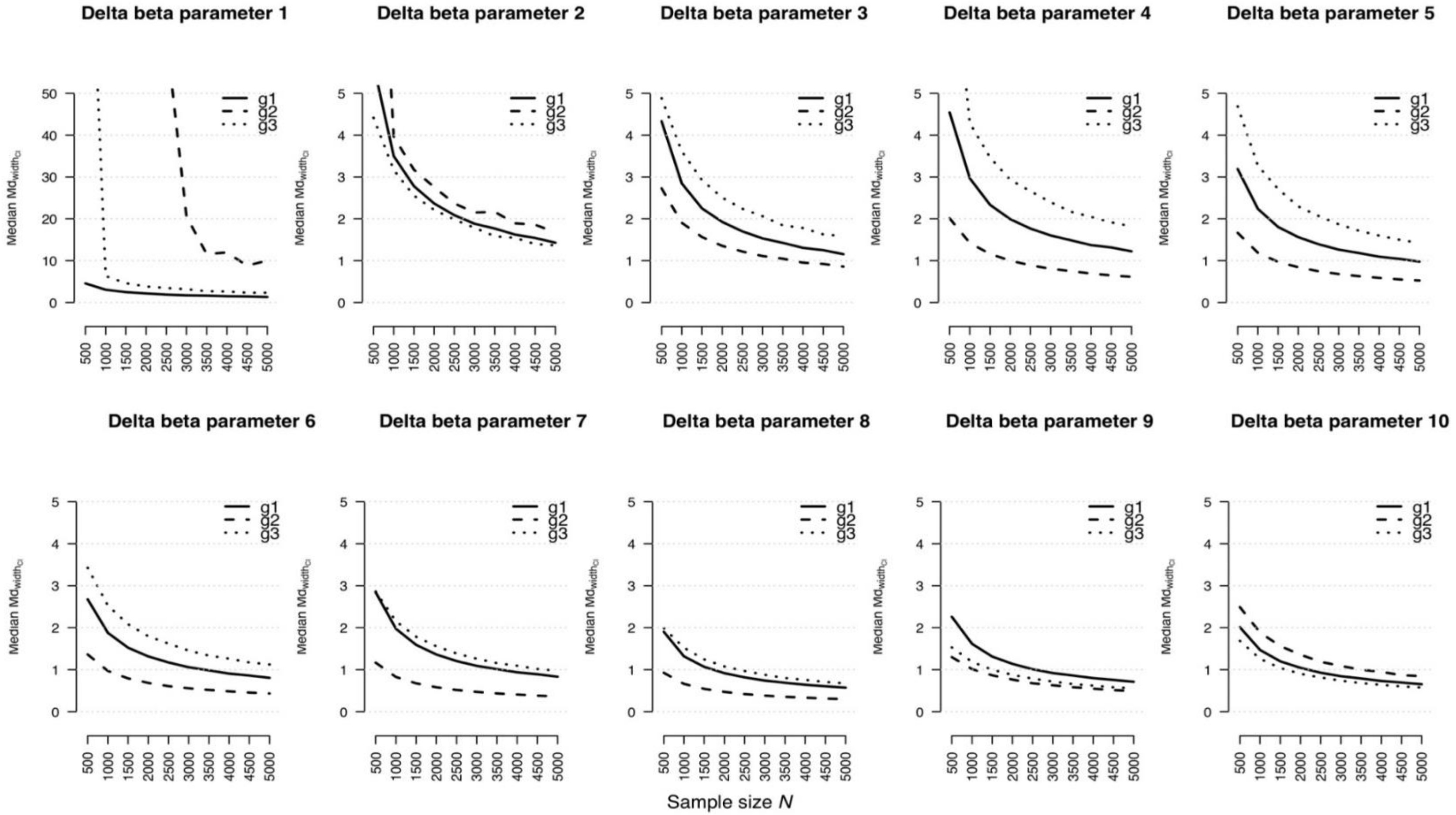

Figure S6. Width of confidence interval for class-specific delta beta parameter estimates in the mPCM-3 *under the condition of a true three-class mixture and a 5-item scale with 11 categories*

Table S7. *Expected category probabilities in latent classes for the population rmGPCM-3 under the condition of a true three-class mixture and a 5-item scale with 11 categories*

		Category										
Item	Class	0	1	2	3	4	5	6	7	8	9	10
1	1	.02	.01	.02	.03	.04	.12	.06	.14	.18	.05	.33
2	1	.02	.01	.02	.02	.02	.06	.02	.04	.11	.05	.61
3	1	.002	.002	.01	.01	.01	.06	.04	.10	.18	.07	.52
4	1	.001	.001	.004	.01	.01	.07	.04	.10	.22	.05	.50
5	1	.004	.003	.01	.01	.01	.07	.02	.07	.14	.05	.61
1	2	.0004	.004	.02	.04	.07	.13	.16	.25	.28	.04	.01
2	2	.001	.01	.02	.04	.04	.08	.08	.15	.31	.16	.13
3	2	0	0	.003	.02	.03	.08	.12	.24	.38	.10	.03
4	2	0	.0001	.003	.02	.04	.13	.16	.30	.31	.03	.01
5	2	.0002	.002	.01	.03	.04	.10	.13	.23	.31	.11	.05
1	3	.004	.02	.03	.05	.04	.09	.09	.21	.21	.23	.03
2	3	.01	.02	.02	.01	.02	.06	.02	.12	.14	.38	.20
3	3	.001	.01	.01	.02	.02	.04	.06	.14	.17	.44	.10
4	3	0	.003	.01	.01	.02	.07	.09	.16	.19	.40	.05
5	3	.002	.01	.01	.02	.03	.07	.05	.10	.17	.37	.18
Mean	1	.01	.01	.01	.02	.02	.08	.04	.09	.16	.06	.52
Mean	2	0	.002	.01	.03	.04	.10	.13	.23	.32	.09	.04
Mean	3	.004	.01	.02	.02	.02	.07	.06	.14	.18	.36	.11

Table S8. *Expected category probabilities in latent classes for the population mPCM-3 under the condition of a true three-class mixture and a 5-item scale with 11 categories*

		Category										
Item	Class	0	1	2	3	4	5	6	7	8	9	10
1	1	.01	.01	.02	.03	.03	.12	.07	.15	.18	.05	.34
2	1	.08	.004	.01	.01	.01	.06	.02	.04	.12	.06	.66
3	1	.002	.002	.01	.01	.01	.06	.04	.09	.18	.07	.54
4	1	.07	.003	.01	.02	.02	.09	.04	.09	.18	.04	.51
5	1	.01	.005	.01	.01	.02	.07	.02	.07	.13	.05	.62
1	2	.001	.004	.02	.04	.06	.13	.16	.26	.27	.05	.01
2	2	.0003	.002	.01	.03	.03	.07	.08	.16	.31	.18	.14
3	2	.0001	.0002	.00	.02	.03	.09	.12	.24	.36	.11	.04
4	2	0	.002	.01	.04	.07	.15	.15	.25	.29	.03	.01
5	2	.001	.01	.02	.05	.05	.11	.12	.21	.28	.11	.04
1	3	.002	.01	.03	.04	.04	.08	.09	.22	.23	.23	.04
2	3	.01	.01	.01	.01	.02	.06	.03	.12	.16	.35	.18
3	3	.001	.01	.01	.01	.01	.04	.05	.14	.18	.46	.10
4	3	.0002	.01	.01	.02	.01	.05	.07	.12	.17	.45	.08
5	3	.004	.01	.01	.01	.02	.06	.04	.08	.15	.39	.22
Mean	1	.01	.004	.01	.02	.02	.08	.04	.09	.16	.05	.53
Mean	2	.000	.003	.01	.03	.05	.11	.13	.22	.30	.10	.05
Mean	3	.002	.01	.02	.02	.02	.06	.06	.14	.18	.38	.12

Table S9. *Convergence rates of the EM algorithm and the Newton-Raphson algorithm, the number of required iterations, and mean classification probability for the rmGPCM and the mPCM under further data conditions*

True latent mixture		Three-class mixture					Two-class mixture				
Condition	*N*	Conv. EM, %	Md_{EM} (Range$_{EM}$)	Conv. NR, %	Md_{NR} (Range$_{NR}$)	$M_{P(Y\mid G)}$	Conv. EM, %	Md_{EM} (Range$_{EM}$)	Conv. NR, %	Md_{NR} (Range$_{NR}$)	$M_{P(Y\mid G)}$
			rmGPCM-3					rmGPCM-2			
15 items 11 cat.	1000	100	141 (87 – 823)	100	9 (7 – 19)	.96	100	100 (53 – 132)	100	10 (8 – 19)	.99
	2500	100	64 (39 – 104)	100	4 (3 – 9)	.95	100	80 (44 – 185)	100	11 (5 – 19)	.99
	4500	100	94 (59 – 165)	100	10 (3 – 15)	.95	100	65 (32 – 102)	100	11 (3 – 17)	.99
5 items 6 cat.	1000	100	335 (122 – 1383)	100	6 (4 – 16)	.85	100	28 (15 – 46)	100	4 (3 – 8)	.95
	2500	100	422 (124 – 2423)	100	5 (3 – 18)	.81	100	23 (10 – 54)	100	4 (3 – 6)	.95
	4500	100	357 (75 – 1002)	100	5 (4 – 12)	.80	100	19 (13 – 34)	100	4 (3 – 12)	.95
15 items 6 cat.	1000	100	98 (45 – 303)	100	4 (2 – 12)	.92	100	33 (18 – 92)	100	4 (3 – 10)	1.00
	2500	100	64 (39 – 104)	100	4 (3 – 9)	.91	100	29 (17 – 78)	100	4 (3 – 9)	1.00
	4500	100	56 (33 – 81)	100	3 (2 – 10)	.91	100	22 (14 – 108)	100	4 (3 – 10)	1.00
			mPCM-3					mPCM-2			
5 items 11 cat.	1000	100	305 (103 – 1415)	100	8 (4 – 419)	.86	100	63 (31 – 248)	100	8 (3 - 12)	.94
	2500	100	154 (88 – 561)	100	7 (3 – 121)	.84	100	40 (26 – 171)	100	5 (3 - 10)	.94
	4500	100	118 (67 – 365)	100	6 (3 – 10)	.83	100	39 (23 – 86)	100	5 (3 – 11)	.93
15 items 11 cat.	1000	100	153 (62 – 586)	100	10 (8 – 16)	.97	100	52 (31 – 112)	100	9 (2 - 15)	.99
	2500	100	60 (46 – 217)	100	7 (3 – 19)	.96	100	39 (28 – 82)	100	5 (2 – 13)	.99.
	4500	100	54 (30 – 134)	100	8 (2 – 14)	.96	100	36 (26 – 56)	100	3 (2 – 18)	.99
5 items 6 cat.	1000	100	288 (118 – 1837)	100	6 (4 – 12)	.84	100	17 (14 – 28)	100	3 (2 – 7)	1.00
	2500	100	468 (91 – 1897)	100	5 (3 – 33)	.80	100	16 (12 –24)	100	3 (2 – 5)	1.00
	4500	100	342 (81 – 2176)	100	5 (3 – 10)	.79	100	15 (12 – 20)	100	3 (2 – 8)	1.00
15 items 6 cat.	1000	100	94 (38 – 671)	100	3 (2 - 9)	.92	100	20 (10 –55)	100	3 (3 – 8)	.95
	2500	100	69 (33 – 121)	100	3 (2 – 7)	.91	100	17 (11 – 22)	100	3 (3 – 5)	.94
	4500	100	60 (28 – 103)	100	3 (2 – 7)	.90	100	15 (11 – 28)	100	3 (3 – 4)	.94

Notes. *N*: sample size condition. Conv.EM: convergence rate of the EM algorithm. Md_{EM} (Range$_{EM}$): median (range) of iterations required to reach a convergent solution of the EM algorithm. Conv.NR: convergence rate of the Newton-Rapson algorithm. Md_{NR} (Range$_{NR}$): median (range) of iterations required to reach a convergent solution of the Newton-Rapson algorithm. $M_{P(Y|G)}$: mean classification probability.

None of the replications produced boundary values. None of the replications were identified as improper solution.

Table S10. *Averaged Spearman's rank correlations between the generating and estimated* $\Delta\beta_{0sg}^{i}$*-parameters for the rmGPCM and the mPCM under further data conditions*

True latent mixture		Three-class mixture						Two-class mixture			
Model		rmGPCM-3			mPCM-3			rmGPCM-2		mPCM-2	
Condition	*N*	g1	g2	g3	g1	g2	g3	g1	g2	g1	g2
5 items 11 cat.	1000							.87	.98	.91	.96
	2500							.98	1.00	.97	1.00
	4500							1.00	1.00	1.00	1.00
15 items 11 cat.	1000	.93	.95	.80	.94	.94	.81	.94	.99	.94	.98
	2500	.99	.99	.93	.99	.99	.93	.99	1.00	1.00	1.00
	4500	1.00	1.00	.98	1.00	1.00	.96	1.00	1.00	1.00	1.00
5 items 6 cat.	1000	1.00	1.00	1.00	1.00	1.00	1.00	1.00	1.00	1.00	1.00
	2500	1.00	1.00	1.00	1.00	1.00	1.00	1.00	1.00	1.00	1.00
	4500	1.00	1.00	1.00	1.00	1.00	1.00	1.00	1.00	1.00	1.00
15 items 6 cat.	1000	1.00	1.00	1.00	1.00	1.00	1.00	1.00	1.00	1.00	1.00
	2500	1.00	1.00	1.00	1.00	1.00	1.00	1.00	1.00	1.00	1.00
	4500	1.00	1.00	1.00	1.00	1.00	1.00	1.00	1.00	1.00	1.00

Note. g1, g2, and g3 indicate three latent classes of two models.

Table S11. *Median root median squared error for parameter estimates (RMdSE) under further data conditions*

True latent mixture	Condition	N	λ^i	$\Delta\beta_{0sg}$	λ_g	π_g	$\Delta\beta_{0sg}$	λ_g	π_g
			rmGPCM-3				mPCM-3		
Three-class mixture	15 items 11 cat.	1000	0.14 [0.06; 0.20]	0.27 [0.13; 0.46]	0.02 [0.01; 0.03]	0.06 [0.03; 0.08]	0.27 [0.13; 0.47]	0.01 [0.01; 0.02]	0.06 [0.03; 0.11]
		2500	0.07 [0.04; 0.13]	0.16 [0.08; 0.28]	0.01 [0.01; 0.03]	0.04 [0.02; 0.06]	0.17 [0.08; 0.28]	0.01 [0.00; 0.01]	0.04 [0.02; 0.06]
		4500	0.06 [0.03; 0.11]	0.12 [0.06; 0.20]	0.01 [0; 0.02]	0.03 [0.02; 0.05]	0.12 [0.06; 0.21]	0.01 [0.00; 0.01]	0.03 [0.01; 0.05]
	5 items 6 cat.	1000	0.27 [0.11; 0.48]	0.31 [0.15; 0.56]	0.05 [0.02; 0.09]	0.33 [0.19; 0.52]	0.29 [0.14; 0.52]	0.04 [0.02; 0.07]	0.25 [0.13; 0.17]
		2500	0.18 [0.08; 0.29]	0.20 [0.11; 0.34]	0.03 [0.01; 0.05]	0.25 [0.12; 0.38]	0.18 [0.09; 0.31]	0.03 [0.01; 0.06]	0.23 [0.13; 0.13]
		4500	0.14 [0.06; 0.27]	0.13 [0.06; 0.22]	0.03 [0.02; 0.04]	0.15 [0.09; 0.25]	0.14 [0.07; 0.23]	0.02 [0.01; 0.04]	0.15 [0.07; 0.13]
	15 items 6 cat.	1000	0.02 [0.01; 0.04]	0.19 [0.10; 0.32]	0.02 [0.01; 0.04]	0.10 [0.04; 0.16]	0.20 [0.10; 0.35]	0.02 [0.01; 0.03]	0.10 [0.05; 0.17]
		2500	0.02 [0.01; 0.02]	0.12 [0.06; 0.20]	0.02 [0.01 0.02]	0.05 [0.02; 0.09]	0.12 [0.06; 0.20]	0.01 [0.00; 0.02]	0.06 [0.02; 0.09]
		4500	0.01 [0; 0.02]	0.09 [0.04; 0.15]	0.01 [0; 0.02]	0.05 [0.02; 0.07]	0.09 [0.04; 0.15]	0.01 [0.00; 0.01]	0.04 [0.02; 0.07]
			rmGPCM-2				mPCM-2		
Two-class mixture	5 items 11 cat.	1000	0.17 [0.07; 0.30]	0.26 [0.11; 0.43]	0.02 [0.01; 0.04]	0.06 [0.02; 0.11]	0.25 [0.11; 0.43]	0.02 [0.01; 0.03]	0.06 [0.02; 0.10]
		2500	0.09 [0.04; 0.19]	0.16 [0.08; 0.28]	0.01 [0.01; 0.02]	0.05 [0.03; 0.08]	0.16 [0.08; 0.28]	0.01 [0.01; 0.02]	0.05 [0.03; 0.08]
		4500	0.07 [0.04; 0.14]	0.13 [0.06; 0.20]	0.01 [0.01; 0.02]	0.04 [0.02; 0.06]	0.12 [0.06; 0.21]	0.01 [0.01; 0.01]	0.03 [0.01; 0.06]
	15 items 11 cat.	1000	0.12 [0.05; 0.19]	0.22 [0.10; 0.37]	0.02 [0.01; 0.03]	0.05 [0.03; 0.09]	0.21 [0.11; 0.36]	0.01 [0; 0.01]	0.06 [0.02; 0.08]
		2500	0.08 [0.04; 0.13]	0.14 [0.07; 0.23]	0.01 [0.01; 0.02]	0.04 [0.01; 0.06]	0.14 [0.07; 0.23]	0.01 [0; 0.01]	0.03 [0.02; 0.05]
		4500	0.05 [0.03; 0.08]	0.11 [0.05; 0.17]	0.01 [0; 0.01]	0.01 [0.01; 0.03]	0.10 [0.05; 0.17]	0.01 [0; 0.01]	0.02 [0.01; 0.04]
	5 items 6 cat.	1000	0.32 [0.15; 0.49]	0.21 [0.10; 0.35]	0.03 [0.02; 0.06]	0.06 [0.04; 0.10]	0.20 [0.11; 0.32]	0.02 [0.01; 0.03]	0.06 [0.03; 0.11]
		2500	0.18 [0.09; 0.32]	0.12 [0.06; 0.20]	0.02 [0.01; 0.04]	0.03 [0.02; 0.06]	0.13 [0.06; 0.20]	0.02 [0.01; 0.02]	0.05 [0.03; 0.08]
		4500	0.14 [0.09; 0.24]	0.09 [0.04; 0.15]	0.02 [0.01; 0.03]	0.03 [0.02;0.06]	0.10 [0.05; 0.17]	0.01 [0.01; 0.02]	0.03 [0.01; 0.05]
	15 items 6 cat.	1000	0.16 [0.07; 0.27]	0.16 [0.08; 0.28]	0.02 [0.01; 0.04]	0.04 [0.02; 0.07]	0.17 [0.08; 0.29]	0.01 [0.01; 0.02]	0.04 [0.01; 0.07]
		2500	0.13 [0.07; 0.21]	0.11 [0.05; 0.18]	0.02 [0.01; 0.03]	0.03 [0.01; 0.05]	0.10 [0.05; 0.17]	0.01 [0; 0.01]	0.03 [0.02; 0.06]
		4500	0.09 [0.04; 0.14]	0.08 [0.04; 0.13]	0.01 [0.01; 0.02]	0.02 [0.01; 0.05]	0.08 [0.04; 0.13]	0.01 [0; 0.01]	0.02 [0.01; 0.04]

Note. The first and the third quartiles are reported in square brackets.

Table S12. *Median bias of standard error estimates (bias$_{se}$) under further data conditions*

True class mixture	Condition	N	λ^i	$\Delta\beta_{0sg}$	λ_g	π_g	$\Delta\beta_{0sg}$	λ_g	π_g
			rmGPCM-3				mPCM-3		
Three-class mixture	15 items 11 cat.	1000	0.02 [0.01; 0.04]	0.05 [0.02 0.12]	0 [0; 0]	0.01 [0; 0.01]	0.05 [0.02; 0.14]	0 [0; 0]	0 [0; 0]
		2500	0.01 [0; 0.02]	0.02 [0.01; 0.04]	0 [0; 0]	0 [0; 0]	0.02 [0.01; 0.05]	0 [0; 0]	0.01 [0.01; 0.01]
		4500	0.01 [0; 0.02]	0.01 [0.01; 0.03]	0 [0; 0]	0 [0; 0.01]	0.02 [0.01; 0.03]	0 [0; 0]	0 [0; 0]
	5 items 6 cat.	1000	0.13 [0.06; 0.31]	0.53 [0.13; 1.12]	0.02 [0.01; 0.06]	0.28 [0.23; 0.32]	0.57 [0.12; 1.07]	0.03 [0.00; 0.04]	0.22 [0.19; 0.27]
		2500	0.04 [0.02; 0.10]	0.08 [0.03; 0.48]	0.01 [0; 0.05]	0.12 [0.06; 0.17]	0.07 [0.03; 0.40]	0.02 [0.01; 0.04]	0.16 [0.08; 0.20]
		4500	0.03 [0.02; 0.06]	0.03 [0.01; 0.07]	0.01 [0; 0.01]	0.04 [0.02; 0.06]	0.03 [0.01; 0.09]	0.01 [0.00; 0.02]	0.08 [0.04; 0.13]
	15 items 6 cat.	1000	0.05 [0.02; 0.08]	0.04 [0.02; 0.07]	0 [0; 0]	0.02 [0.01; 0.04]	0.04 [0.02; 0.09]	0 [0; 0]	0.03 [0.02; 0.04]
		2500	0.02 [0.01; 0.04]	0.02 [0.01; 0.03]	0 [0; 0]	0 [0; 0.01]	0.02 [0.01; 0.03]	0 [0; 0.01]	0.01 [0.01; 0.01]
		4500	0.01 [0.01; 0.02]	0.01 [0.01; 0.02]	0 [0; 0]	0.01 [0; 0.01]	0.01 [0; 0.02]	0 [0; 0]	0.01 [0; 0.01]
			rmGPCM-2				mPCM-2		
Two-class mixture	5 items 11 cat.	1000	0.04 [0.02; 0.08]	0.07 [0.02; 0.84]	0.01 [0; 0.01]	0.01 [0; 0.01]	0.07 [0.02; 0.74]	0 [0; 0]	0.01 [0.01; 0.02]
		2500	0.01 [0.01; 0.03]	0.03 [0.01; 0.06]	0 [0; 0]	0.01 [0; 0.01]	0.02 [0.01; 0.06]	0 [0; 0]	0.01 [0.01; 0.01]
		4500	0.01 [0; 0.02]	0.01 [0.01; 0.03]	0 [0; 0]	0.01 [0.01; 0.01]	0.02 [0.01; 0.04]	0 [0; 0]	0 [0; 0]
	15 items 11 cat.	1000	0.02 [0.01; 0.04]	0.03 [0.01; 0.09]	0 [0; 0]	0.01 [0.01; 0.01]	0.03 [0.01; 0.10]	0 [0; 0]	0.01 [0.01; 0.01]
		2500	0.01 [0.01; 0.02]	0.02 [0.01; 0.04]	0 [0; 0]	0 [0; 0]	0.02 [0.01; 0.04]	0 [0; 0]	0.01 [0.01; 0.01]
		4500	0.01 [0; 0.01]	0.01 [0; 0.03]	0 [0; 0]	0.01 [0.01; 0.01]	0.01 [0; 0.02]	0 [0; 0]	0 [0; 0]
	5 items 6 cat.	1000	0.11 [0.07; 0.21]	0.04 [0.01; 0.10]	0 [0; 0.01]	0 [0; 0.01]	0.03 [0.01; 0.10]	0 [0; 0.01]	0.01 [0.01; 0.01]
		2500	0.07 [0.04; 0.13]	0.01 [0.01; 0.03]	0 [0; 0]	0 [0; 0]	0.01 [0.01; 0.04]	0 [0; 0]	0.01 [0; 0.01]
		4500	0.02 [0.01; 0.04]	0.01 [0; 0.02]	0 [0; 0]	0.01 [0.01; 0.01]	0.01 [0; 0.02]	0 [0; 0]	0 [0; 0]
	15 items 6 cat.	1000	0.04 [0.02; 0.07]	0.02 [0.01; 0.05]	0.01 [0; 0.01]	0 [0; 0.01]	0.02 [0.01; 0.04]	0 [0; 0]	0.01 [0.01; 0.01]
		2500	0.03 [0.01; 0.05]	0.01 [0; 0.02]	0 [0; 0]	0 [0; 0]	0.01 [0.01; 0.02]	0 [0; 0]	0 [0; 0]

4500	0.01 [0.01; 0.02]	0.08 [0; 0.02]	0 [0; 0]	0 [0; 0.01]	0.01 [0; 0.01]	0 [0; 0]	0 [0; 0]

Note. The first and the third quartiles are reported in square brackets.

Table S13. *Median width of confidence interval for parameter estimates (width$_{CI}$) under further data conditions*

True latent mixture	Condition	N	λ^i	$\Delta\beta_{0sg}$	λ_g	π_g	$\Delta\beta_{0sg}$	λ_g	π_g
				rmGPCM-3				mPCM-3	
Three-class mixture	15 items 11 cat.	1000	0.72 [0.51; 1.00]	1.46 [1.02; 2.12]	0.09 [0.09; 0.10]	0.33 [0.32; 0.36]	1.48 [1.01; 2.22]	0.06 [0.06; 0.07]	0.33 [0.30; 0.36]
		2500	0.43 [0.31; 0.59]	0.93 [0.66; 1.35]	0.06 [0.06; 0.06]	0.21 [0.20; 0.23]	0.94 [0.65; 1.43]	0.04 [0.04; 0.05]	0.21 [0.19; 0.23]
		4500	0.32 [0.24; 0.44]	0.69 [0.49; 1.00]	0 .04 [0.04; 0.05]	0.16 [0.15; 0.17]	0.70 [0.49; 1.06]	0.03 [0.03; 0.04]	0.16 [0.14; 0.17]
	5 items 6 cat.	1000	1.70 [1.07; 2.63]	1.50 [1.08; 2.25]	0.22 [0.19; 0.27]	0.81 [0.55; 1.08]	1.50 [1.09; 2.20]	0.18 [0.14; 0.27]	0.85 [0.59; 1.16]
		2500	0.99 [0.69; 1.44]	1.02 [0.76; 1.39]	0.15 [0.12; 0.20]	0.91 [0.65; 1.28]	0.98 [0.75; 1.34]	0.13 [0.09; 0.19]	0.87 [0.61; 1.10]
		4500	0.74 [0.52; 1.17]	0.74 [0.57; 0.99]	0.11 [0.09; 0.13]	0.82 [0.59; 0.96]	0.75 [0.57; 1.03]	0.11 [0.07; 0.14]	0.79 [0.58; 1.07]
	15 items 6 cat.	1000	1.06 [0.77; 1.44]	1.07 [0.87; 1.43]	0.14 [0.13; 0.15]	0.44 [0.40; 0.50]	1.11 [0.88; 1.45]	0.09 [0.07; 0.10]	0.45 [0.37; 0.54]
		2500	0.66 [0.48; 0.90]	0.67 [0.55; 0.89]	0.09 [0.08; 0.09]	0.28 [0.25; 0.32]	0.70 [0.57; 0.89]	0.05 [0.05; 0.06]	0.29 [0.24; 0.35]
		4500	0.48 [0.36; 0.63]	0.50 [0.41; 0.66]	0.07 [0.06; 0.07]	0.21 [0.18; 0.24]	0.52 [0.43; 0.66]	0.04 [0.03; 0.05]	0.22 [0.18; 0.26]
				rmGPCM-2				mPCM-2	
Two-class mixture	5 items 11 cat.	1000	1.05 [0.67; 1.56]	1.51 [0.93; 2.50]	0.12 [0.12; 0.13]	0.40 [0.39; 0.42]	1.47 [0.91; 2.50]	0.09 [0.08; 0.10]	0.39 [0.38; 0.41]
		2500	0 .63 [0.41; 0.91]	0.95 [0.59; 1.54]	0.08 [0.08; 0.08]	0.26 [0.25; 0.27]	0.92 [0.57; 1.55]	0.06 [0.05; 0.07]	0.25 [0.25; 0.26]
		4500	0.48 [0.34; 0.74]	0.70 [0.44; 1.13]	0.06 [0.06; 0.06]	0.19 [0.19; 0.20]	0.68 [0.43; 1.14]	0.04 [0.04; 0.05]	0.19 [0.19; 0.19]
	15 items 11 cat.	1000	0.68 [0.49; 0.98]	1.25 [0.83; 1.97]	0.09 [0.09; 0.10]	0.27 [0.27; 0.27]	1.24 [0.82; 2.01]	0.05 [0.05; 0.06]	0.27 [0.27; 0.27]
		2500	0.44 [0.32; 0.61]	0.79 [0.52; 1.26]	0.06 [0.06; 0.06]	0.17 [0.17; 0.17]	0.78 [0.52; 1.28]	0.04 [0.03; 0.04]	0.17 [0.17; 0.17]
		4500	0.32 [0.24; 0.43]	0.59 [0.39; 0.94]	0.04 [0.04; 0.05]	0.13 [0.13; 0.13]	0.58 [0.39; 0.95]	0.03 [0.02; 0.03]	0.13 [0.13; 0.13]
	5 items 6 cat.	1000	1.60 [1.09; 2.54]	1.18 [0.58; 1.69]	0.19 [0.17; 0.21]	0.32 [0.33; 0.35]	1.18 [0.57; 1.65]	0.12 [0.12; 0.13]	0.35 [0.34; 0.36]
		2500	1.02 [0.74; 1.53]	0.75 [0.37; 0.99]	0.12 [0.11; 0.13]	0.22 [0.21; 0.22]	0.73 [0.37; 1.00]	0.08 [0.07; 0.08]	0.22 [0.22; 0.23]
		4500	0.73 [0.50; 1.09]	0.56 [0.27; 0.75]	0.09 [0.08; 0.10]	0.16 [0.16; 0.16]	0.55 [0.27; 0.75]	0.08 [0.07; 0.06]	0.17 [0.16; 0.17]
	15 items 6 cat.	1000	1.05 [0.79; 1.47]	0.99 [0.53; 1.35]	0.13 [0.13; 0.14]	0.27 [0.27; 0.27]	0.99 [0.52; 1.34]	0.06 [0.06; 0.07]	0.27 [0.27; 0.27]
		2500	0.66 [0.49; 0.90]	0.62 [0.33; 0.85]	0.09 [0.08; 0.09]	0.17 [0.17; 0.17]	0.62 [0.33; 0.85]	0.04 [0.04; 0.05]	0.17 [0.17; 0.17]

4500	0.48 [0.35; 0.63]	0.46 [0.25; 0.63]	0.06 [0.06; 0.07]	0.13 [0.13; 0.13]	0.46 [0.25; 0.63]	0.03 [0.03; 0.03]	0.13 [0.13; 0.13]

Note. The first and the third quartiles are reported in square brackets.
